# An analytical four-layer horizontal electric current dipole model for analysing underwater electric potential in shallow seawater

**DOI:** 10.1038/s41598-022-12645-z

**Published:** 2022-05-24

**Authors:** Miroslaw Woloszyn, Krystian Buszman, Tomasz Rutkowski, Jaroslaw Tarnawski, Francisco Javier Rodrigo Saura

**Affiliations:** 1 Faculty of Electrical and Control Engineering, Gdansk University of Technology, Gdansk, Poland; 2 Faculty of Navigation and Naval Weapons, Polish Naval Academy, Gdynia, Poland; 3 SAES-Sociedad Anónima de Electrónica Submarina, Cartagena, Spain

**Keywords:** Engineering, Electrical and electronic engineering

## Abstract

The paper presents a new analytical four-layer (air–water–bottom–non-conductive layer) horizontal electric dipole model which allows an accurate approximation of ship's Underwater Electric Potential (UEP) from a sufficient depth in shallow coastal marine waters. The numerical methods, usually Finite Element Method (FEM) or Boundary Elements Method (BEM), are typically used to estimate the electric field and the distribution of static electric components of UEP around the ship. These methods enable analyses with high accuracy but, compared to other point-electrode methods and the proposed analytical model, they are relatively complex and need high computational time. The developed analytical model proposed in this paper allows real-time calculations without significant loss of accuracy of the UEP estimations. In the model, the problem of boundary values at the borders of individual layers is solved using the reflection/image method and applying the idea of continuity of electric potential at a given boundary between two adjacent layers. Its accuracy is verified based on the synthetic data provided by specialised software packages making use of FEM and BEM numerical methods. A dimensionless quantitative analysis of the relationships between basic parameters of the proposed four-layer analytical model and their impact on the accuracy of representation of individual electric field strength components is also delivered. The relationships between water and bottom conductivity and between water depth and bottom thickness are investigated and described. The obtained results show that the developed model allows detailed and reliable analysis of the electric field, especially in shallow coastal waters.

## Introduction

For years, electric field related technologies have been of interest in various fields of science and technology such as geophysics, archaeology, industrial and chemical process monitoring, and biomedical engineering. Meaningful applications of these technologies may be found in systems used in Electrical Impedance Tomography (EIT)^1,2^, Electrical Resistivity Tomography (ERT)^3^, or Induced Polarization Tomography (IPT)^4^. In such applications, currents with a sinusoidal or rectangular waveform are usually applied to the analysed object. The resulting voltages are measured using the surface electrodes to assess the interior conductivity distribution and the permittivity distribution. Not only man-made technical devices use electric field technologies. For example, in the natural world, similar techniques are used by weakly electric fish. For food acquisition and navigation purposes, those fishes can generate an electric field and use echo analysis with receptors placed on their bodies. This natural phenomenon, called biological electrosense, is extensively studied by numerous researchers. For example, mathematical models for weakly electric fish in terms of active electrolocation can be found in^5^, while those concerning shape recognition and classification in^6^. Advanced optimization or discontinuity testing methods are used in science to find the structure and parameters of such models. However, it is hard to expect living organisms to act in this way. The papers^7^ and^8^ present a simplified approach and mathematical framework in the form of first-order polarization tensor for the purpose of object electrosensing, while^9^ shows the implementation of an algorithm for estimating the size, shape, orientation, and location of an ellipsoidal object at short range in the robotic active electrosense system.

In this article, the authors focus on applying underwater electrical potential techniques in marine industry. The measurement of underwater electrical potential (UEP) is widely used in civil and military marine applications for different purposes. For example, it is used for monitoring a ship hull corrosion and its protection against corrosion^10–15^, in predicting UEP signatures to evaluate a possible risk of the vessel beaning detected by naval mines^16^, in geophysical surveys to obtain the information on seabed structure^17,18^, in the exploration of oil and gas deposits located in the seafloor^19,20^ in the tasks of ships tracking^21^ and object localization below the waterline^22^, or in ships classification^23^.

The electric signature of maritime platforms is a measure of the static electric field generated by the electric currents flow around the vessel's hull. These currents are generated by galvanic corrosion of the hull (natural ship corrosion state) or, as anti-corrosion currents, by Impressed Current Cathodic Protection (ICCP) systems or passive protection systems based on the usage of sacrificial anodes, designed to prevent corrosion (cathodic ship protection state). The currents flow between particular ship's hull components made of different electrochemical materials when they are immersed in seawater. In this case, the seawater acts as an electrolyte, and the electric circuit is closed through the metallic hull of the ships or even through its internal cabling system. The source electrode (anode) in this circuit is the submerged part of the ship's hull and sacrificial anodes of the passive cathodic systems, while the sink electrodes (cathode) are the shaft propellers and hull damages. From a sufficient depth, this phenomenon can be approximated by a single horizontal electric current dipole system^24^. Along with magnetic anomalies and hydrodynamic variations detected in the presence of the ship, the static electric component of the electric field is one of physical fields referred to as ship signatures^25,26^. The authors have experience in analyzing and modeling the magnetic field distribution with the use of a multi-dipole model for both synthetic data^27,28^ and real data from measurements^29^.

The ship's UEP signatures depend on the type and state of the used materials, their locations along the ship and the size of the galvanic components installed on the ship's hull, which constitute the object-specific configuration of anodes and cathodes. For a ship equipped with cathodic protection, its UEP signature is much more significant (in terms of value) and complex (number of peaks) due to the presence of additional anodes and currents generated by external power systems. The UEP signatures are of particular importance for the safety of ships triggering sea mines or other vessel detection systems (typically located close to the bottom) equipped with integrated electric field sensors, which are characterised by high sensitivity and low measurement drift and enable UEP monitoring over a wide frequency range^25^. Hence, to decrease the ship’s electric field, the process of optimizing its structure is used, along with the impressed current compensation^30^ and advanced ICCP systems^10,11^. The authors of the paper^31^ compared those methods and showed that the impressed current compensation method should be used to achieve the maximum reduction of the ship’s static electric field signal.

**Figure 1:**
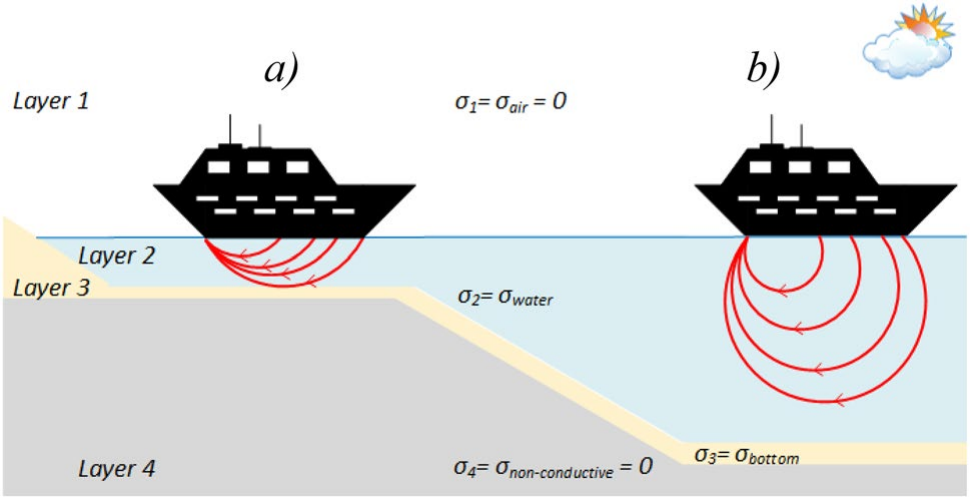
The four-layer model structure: layer 1—air, layer 2—water, layer 3—bottom, layer 4—non-conductive layer. Red arrows denote: (**a**) characteristic flattening of the electric field at layer 3 boundary without deep penetration into the bottom layer 4 (shallow water), (**b**) typical electric field at the deep-water area.

This paper presents and verifies, using synthetic data as the reference, a new analytical four-layer (air–water–bottom–non-conductive layer) horizontal electric dipole model—Fig. 1, which allows an accurate approximation of the ship's underwater electric potential UEP in shallow coastal marine waters, the bottom of which may have different structure (bottom layer thickness) and may be characterized by different parameters (bottom and water layer conductivities—*σ*_*2*_ and *σ*_*3*_, respectively). Until now, numerous authors have already described two-layer and three-layer models of the horizontal current dipole but in those cases they had to adapt appropirelly the models parametres to handle the bottomsea characteristics^24,32^. While, according to the best of authors' knowledge, the four-layer model has not yet been presented in the literature.

In shallow water and nearby seabed, due to conductivity difference between water and bottom, a specific reflection of the electric field takes place on the boundary between these two media^24,33–35^. Additionally, when considering the fourth non-conductive layer with a particular thickness below the bottom, characteristic flattening of the electric field at layer’s boundary without deep penetration into the bottom layer is observed—Fig. 1. Typically, the sea/ocean floor in coastal waters is covered with sediments resulting from the decomposition of organic matter and particles of inorganic matter deposited by rivers, or even volcanic ash fall^36^. Depending on sea currents, this layer of sediments can vary in thickness, and in addition, depending on the dominant material, it can have different electrical conductivity, which is strongly related to the distribution of the electric field generated by the ship. Furthermore, the mixing of seawater with freshwater, e.g. in river deltas, affects the chemistry of the seawater, which can additionally influence the potential recorded by electric sensor electrodes^24,35^.

Specialised software packages, such as OPERA^37^, SIMSEN^38^ from SAES^39^, BEASY Corrosion^40^, or COMSOL Multiphysics^41^, which make use of Finite Element Method (FEM) or Boundary Elements Method (BEM) methods, are typically used to model the electric field distribution and the UEP signature around the ship. These methods enable analyses with high accuracy^42^ and can be used to estimate the electric filed of a maritime platform from the definition phase up to the sea trials. However, compared to other point-electrode methods and the model presented in this paper, they are relatively complex and need high computational time^42^. At the same time, the proposed four-layer model enables real-time calculations without significant deterioration the accuracy of the obtained results. In this model, the problem of boundary values at the borders between individual layers is solved using the "reflection/image" method and applying the idea of continuity of electric potential at a given boundary between two adjacent layers.

In^32^, the author has described the electromagnetic field generated by a horizontal electric dipole in the three-layer region. The complex equations field formulas included all points in all three layers. The model of electric dipole and the inversion method were used to get an equivalent ship's electric field. The authors of the paper^42^ have presented the ships' UEP model based on the multiple point-electrode method which allowed reconstructing more precisely ship’s electric signature close to the hull than the single point-electrode (single dipole) method. They used the Particle Swarm Optimization (PSO) method to solve the number, positions, and current values of point-electrodes according to the UEP distribution at a known depth below the keel of a ship. Based on the scaled ship model experiment, the authors have shown that the resulting model can be used for simulation and prediction of UEP signatures.

The accuracy of the four-layer analytical model proposed in this paper was verified based on the synthetic data obtained using specialised software packages (OPERA^37^, SAES^38,39^) making use of FEM and BEM numerical methods, respectively, to model a single horizontal current dipole in the marine environment with shallow water (Fig. 1). Furthermore, based on the analytical studies, it was also indicated how to select the number of "reflections/images" depending on the width of the water layer to maintain satisfactory quality of electric field intensity reproduction (< 1%). A dimensionless quantitative analysis of the relationships between basic parameters of the proposed four-layer analytical model and their impact on the quality of representation of individual electric field strength components was also performed. The relationships between water and bottom conductivity and between water depth and bottom thickness were investigated and described.

The analysis of the electric field generated by an electric current dipole for many scenarios has shown that the fourth layer in the proposed analytical model is significant in the distribution of the electric field in seawater, especially when the bottom thickness is small and/or the electric conductivity of the bottom is much lower than that of seawater. Furthermore, the results presented and described allow the authors to conclude that the proposed four-layer analytical model reproduces the electrical signature with satisfactory quality in shallow coastal and deep-water areas.

The paper is organized as follows. “Analytical model of four-layer horizontal electric current dipole” presents the description of the proposed analytical model of four-layer horizontal electric current dipole. “Verification of the analytical four-layer model—comparing to numerical FEM and BEM models” reports the verification of the proposed model by comparing it to numerical models built using the finite element method (FEM) and the boundary element method (BEM). “Analytical four-layer model-based analysis of electric field” gives the results of the dimensionless quantitative analysis of the relationships between basic parameters of the proposed four-layer analytical model and their impact on the quality of representation of individual electric field strength components. Finally, “Conclusions” presents conclusions of the paper.

## Analytical model of four-layer horizontal electric current dipole

### Fundamental equations

The electric field intensity **E** for an irrotational field is defined in terms of the existing scalar electric potential *V*, as follows^43^

1 $$\mathbf{E}=-\nabla V.$$

Additionally, the fundamental law of current conservation is expressed in Maxwell’s notation as^43^

2 $$\nabla \cdot \mathbf{J}=-\frac{\partial \rho }{\partial t},$$

where $\mathbf{J}$ denotes the current density. For direct currents (DC):

3 $$\frac{\partial \rho }{\partial t }=0,$$

therefore

4 $$\nabla \cdot \mathbf{J}=0.$$

According to Ohm’s law, the current density **J** is proportional to the electric field **E**,^43^

5 $$\mathbf{J}=\sigma \mathbf{E},$$

where $\sigma$ denotes the electric conductivity. Hence, Eq. (4), which can be rewritten to the form

6 $$\nabla \cdot \left(\sigma \mathbf{E}\right)=-\nabla \cdot (\sigma \nabla V)=0,$$

satisfies Laplace’s equation

7 $${\nabla }^{2}V=0.$$

There are an infinite number of functions that satisfy Laplace's Eq. (7), and for a given case, the unique solution is found by specifying appropriate boundary conditions. Once the electrostatic potential *V* has been calculated, the components of electric field intensity *E*, e.g. in the Cartesian coordinate system (*x*, *y, z*), may be computed by taking into account the gradient of the potential *V* in appropriate direction (Eq. 1) and the Ohm’s law expressed by relation (Eq. 5).

### Application of the method of images

In the developed model, the method of images is used to calculate the electric field intensity $\mathbf{E}$ as the solution of Laplace's Eq. (4), along with the boundary conditions between materials or media are needed to determine the solution uniquely. It is known that the tangential component of the electric field intensity *E* on the surface of a conductor is equal to zero, and its total value in the considered region is uniquely defined by its normal component over the surface that confines this region^43^. Hence, the boundary conditions in the area between two considered materials or media with electric conductivities *σ*_1_ and *σ*_2_ can be expressed as

8 $${j}_{n1}={j}_{n2}$$

9 $${E}_{t1}= {E}_{t2} \Leftrightarrow \frac{{j}_{t1}}{{\sigma }_{1}}=\frac{{j}_{t2}}{{\sigma }_{2}}$$

where ${j}_{n1}, {j}_{n2}$ denote the normal components of steady current sources on the boundary between two materials/media 1 and 2, while ${j}_{t1},{j}_{t2}$ denote the tangential components of current densities on the boundary between two materials/media 1 and 2*,* respectively.

**Figure 2:**
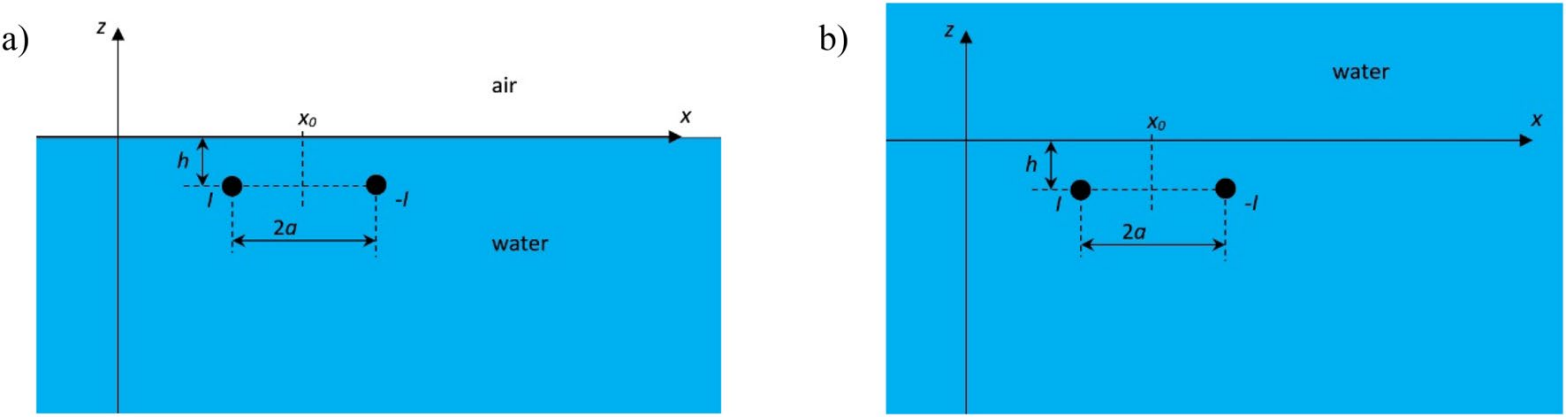
Electric electrodes in seawater: (**a**) two layers: air and water; (**b**) one layer: water.

In order to derive the analytical model of the electric current dipole and calculate its underwater electric potential, let us consider two spherical electrodes with current *I*, which are placed at depth *h* below the water surface (Fig. 2a). In Fig. 2a, *x*_*0*_ denotes the displacement of the electrodes (along the *x*-axis) relative to the center of the Cartesian coordinate system, *h* represents the depth of their placement, and *2a* denotes the distance between the electrode with current *I* (positive) and that with current − *I* (negative).

For the clarity of further considerations, let us first present the basic equations for the distribution of the electric potential *V*_*I*_ and individual components *E*_*x*_, *E*_*y*_ and *E*_*z*_ of the electric field intensity only in the water layer (case shown in Fig. 2b). Notice that it is unnecessary to use the mirror method in this case, and the components *E*_*x*_, *E*_*y*_ and *E*_*z*_ can be calculated directly from the equations presented below. Hence, the potential at any point in the Cartesian coordinate system is calculated from the equation:

10 $${V}_{I}\left(x,y,z\right)=\frac{1}{4\pi {\sigma }_{w}}\left(\frac{I}{\sqrt{{(x-{x}_{0}-a)}^{2}+{y}^{2}{+(z-h)}^{2}}}+\frac{-I}{\sqrt{{(x-{x}_{0}+a)}^{2}+{y}^{2}{+(z-h)}^{2}}}\right).$$

whereas the individual electric field intensity components are calculated based on Eq. (2), which in this case can take the following form

11 $${E}_{x,I}= -\frac{\partial {V}_{I}\left(x,y,z\right)}{\partial x}=-\frac{I}{4\pi {\sigma }_{w}}\left(\frac{-\left(x-{x}_{0}-a\right)}{{{((x-{x}_{0}-a)}^{2}+{y}^{2}{+\left(z-h\right)}^{2})}^{3/2}}+\frac{\left(x-{x}_{0}+a\right)}{{{((x-{x}_{0}+a)}^{2}+{y}^{2}{+\left(z-h\right)}^{2})}^{3/2}}\right),$$

12 $${E}_{y,I}= -\frac{\partial {V}_{I}\left(x,y,z\right)}{\partial y}=-\frac{I}{4\pi {\sigma }_{w}}\left(\frac{-y}{{{((x-{x}_{0}-a)}^{2}+{y}^{2}{+\left(z-h\right)}^{2})}^{3/2}}+\frac{y}{{{((x-{x}_{0}+a)}^{2}+{y}^{2}{+\left(z-h\right)}^{2})}^{3/2}}\right),$$

13 $${E}_{z,I}= -\frac{\partial {V}_{I}\left(x,y,z\right)}{\partial z}=-\frac{I}{4\pi {\sigma }_{w}}\left(\frac{-\left(z-h\right)}{{{((x-{x}_{0}-a)}^{2}+{y}^{2}{+\left(z-h\right)}^{2})}^{3/2}}+\frac{\left(z-h\right)}{{{((x-{x}_{0}+a)}^{2}+{y}^{2}{+\left(z-h\right)}^{2})}^{3/2}}\right).$$

**Figure 3:**
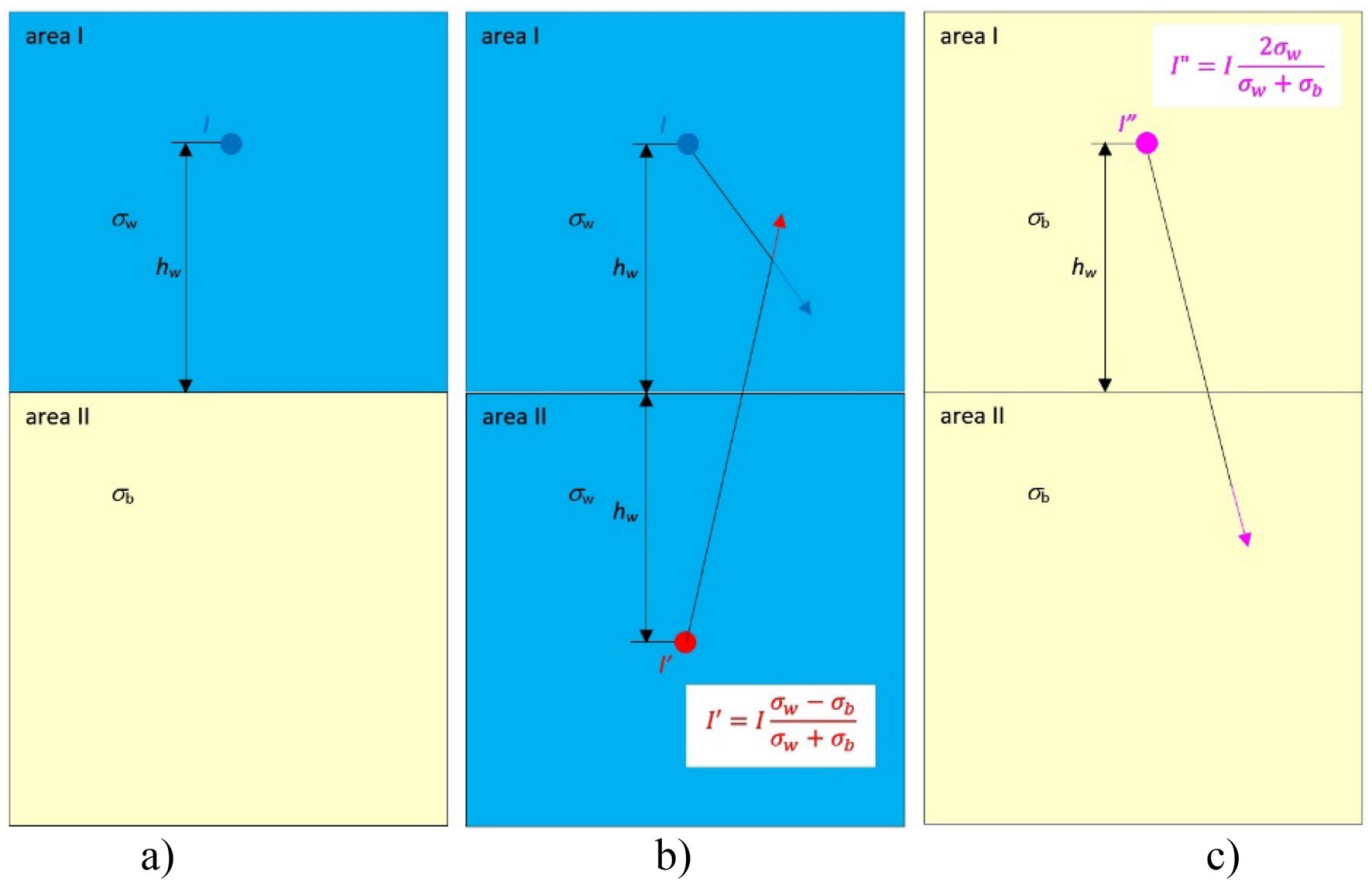
Method of electric field calculation in two layers with different electric conductivities.

When the current electrode *I* is located in area I (Fig. 3a) with electric conductivity *σ*_w_ which is situated next to area II with electric conductivity *σ*_*b*_, two additional virtual current electrodes *I*′ and *I*″ (taking into account the axial symmetry of the border between those areas) should be introduced to solve the electric field in those two areas. The virtual electrode *I*′ is introduced in area II (Fig. 3b), and the virtual electrode *I*″ in area I (Fig. 3c). The real current electrode *I* and the virtual electrode with current *I*′ reflected at a distance 2*h*_w_ from the real electrode allow calculating the electric field in the space which is only valid in area I (Fig. 3b). At the same time, the virtual electrode *I*″ located at the same position as the real electrode (area I) allows to calculate the electric field in the space which is only valid in area II with electric conductivity *σ*_*b*_ (Fig. 3c).

Through analyzing where the new virtual electrodes with currents *I*, *I*′ and *I’’* have been introduced and how they interact with the corresponding layers (Fig. 3a–c), the following equations must be satisfied to guarantee meeting the boundary conditions BC1, BC2 and BC3 on the defined boundaries

14 $$I - I^{\prime} = I^{\prime\prime},$$

15 $$\frac{I}{{\sigma_{{\text{w}}} }} + \frac{{I^{\prime}}}{{\sigma_{{\text{w}}} }} = \frac{{I^{\prime\prime}}}{{\sigma_{{\text{b}}} }}$$

After solving this set of equations, the following formulas for virtual sources of currents *I′* and *I″* are obtained

16 $$I^{\prime} = I\frac{{\sigma_{w} - \sigma_{b} }}{{\sigma_{w} + \sigma_{b} }},$$

17 $$I^{\prime\prime} = I\frac{{2\sigma_{b} }}{{\sigma_{w} + \sigma_{b} }}.$$

The above methodology should also be applied to the negative electrode to fully describe the considered case (Fig. 2a).

**Figure 4:**
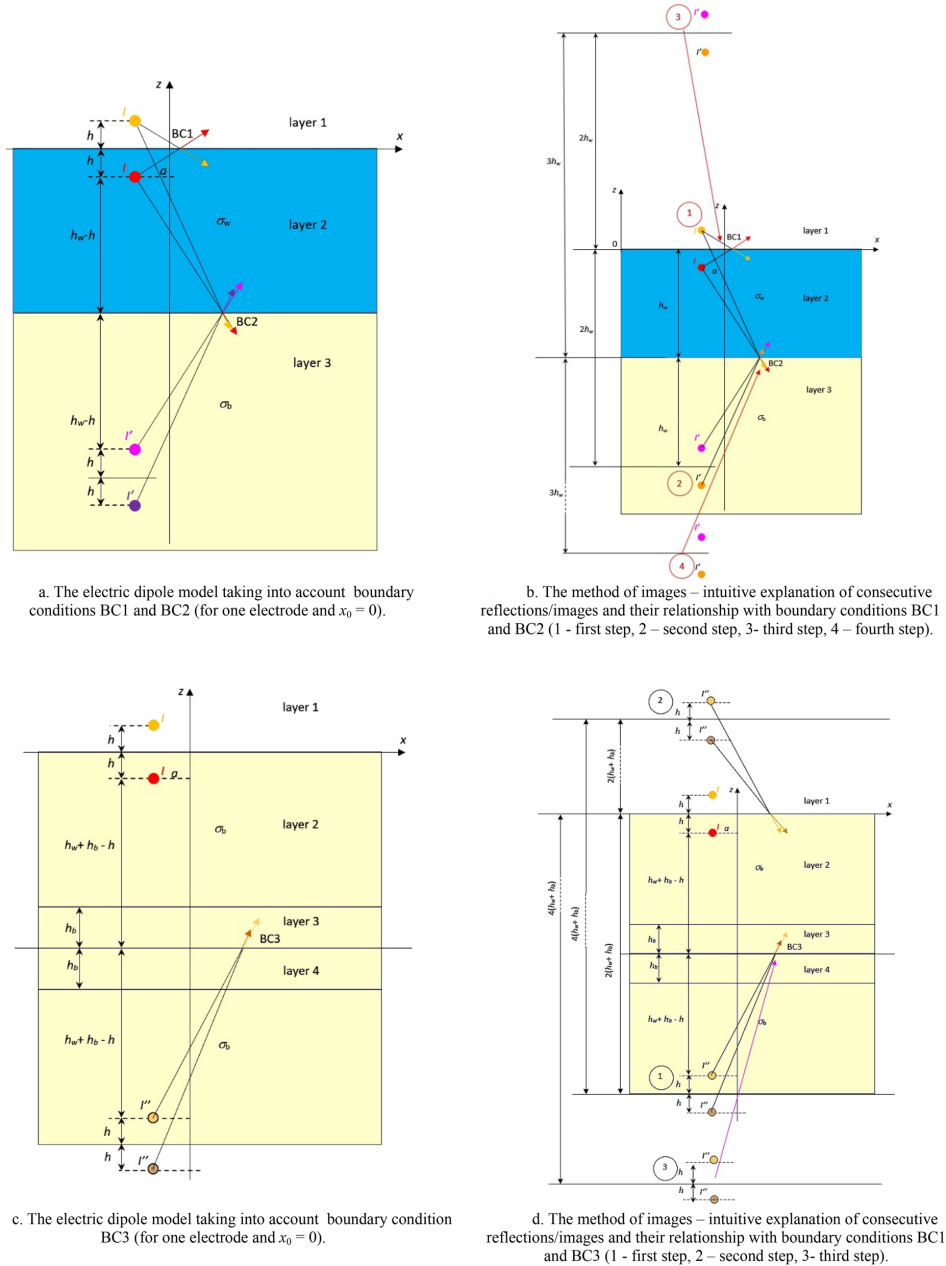
(**a**) The electric dipole model taking into account boundary conditions BC1 and BC2 (for one electrode and *x*_0_ = 0). (**b**) The method of images—intuitive explanation of consecutive reflections/images and their relationship with boundary conditions BC1 and BC2 (1—first step, 2—second step, 3—third step, 4—fourth step). (**c**) The electric dipole model taking into account boundary condition BC3 (for one electrode and *x*_0_ = 0). (**d**) The method of images—intuitive explanation of consecutive reflections/images and their relationship with boundary conditions BC1 and BC3 (1—first step, 2—second step, 3—third step).

When considering a more complex model with two layers (Fig. 2a), or the proposed in the paper model with four layers (Fig. 1), it is necessary to consider conditions at the boundaries between all layers defined in the model. In the former case, it will be only one boundary condition BC1 between air layer and water layer, while the latter case will require three boundary conditions: BC1 between air layer and water layer, BC2 between water layer and bottom layer, and BC3 between bottom layer and non-conductive layer (Fig. 4a–d). It is assumed that the water depth and the bottom thickness are equal to *h*_*w*_ and *h*_*b*_, respectively.

Now let us consider in detail the four-layer model (Fig. 1). To take into account the boundary condition BC1 on the water surface (where the normal component of the electric intensity field vector equals zero), according to the method of images a virtual electrode must be introduced over the water surface at height *h* (*z* = *h*) in layer 1—air (Fig. 4a). In the case of two layers (air–water), only the virtual electrode with current *I* above the water surface is needed. In the case of three layers (air–water–seabottom), a pair of virtual electrodes *I*′ has to be introduced to satisfy boundary conditions BC2 (*z* = − 2*h*_w_ underwater − second reflection), but on the other hand these electrodes disturb the boundary condition BC1. Therefore, in the next (third) reflection, a pair of virtual electrodes *I*′ should be introduced at height 2*h*_w_ (*z* =  + 2*h*_w_ above water) Fig. 4b. These electrodes (at point 3) disturb the boundary condition BC2, which means that the successive (fourth) pair of virtual electrodes should be introduced at depth 4*h*_w_ (*z* = − 4*h*_w_ underwater) and the next mirrors ± 2*nh*_w_ (*n—*number of reflections) should be taken into consideration.

The electrodes with currents *I* and *I′* affect layer 2 (water)—as they generate an electric field intensity only in this layer, which is presented in Fig. 4a as yellow, pink and purple arrows. It should be noted that in the presented model, the resulting electric field intensity components are calculated only in layer 2 (water). Hence, it is assumed that the electric sensors are located over the bottom and inside the water layer in this case.

In the case of four layers (air–water–seabottom–nonconducting), the pair of virtual electrodes *I″* has to be introduced to satisfy boundary conditions BC3 (Fig. 4c). To take into account the boundary condition BC3 on the bottom surface, the non-conductive layer where the normal component of the electric field intensity vector equals zero, the virtual electrodes with current *I″* are placed in the non-conductive layer 4—Fig. 4c. The virtual electrodes *I″* shown in Fig. 3c generate the electric field intensity only in the seabottom. This electric field is not shown in Fig. 4c,d because the electric field is calculated only in water. However, the electric field generated by electrodes *I*″ placed in *z* =  ± *h* exists in layer 3, and the vertical component of the electric field intensity in boundary BC3 is not equal to zero. Therefore, the virtual electrodes *I*″ are placed at point *z* = − 2(*h*_w_ + *h*_b_) (first reflection) to satisfy the boundary condition BC3, and at the second reflection point *z* =  + 2(*h*_w_ + *h*_b_) (above water) to meet the boundary condition BC1 (Fig. 4b). The fourth and next mirrors, ± 2*i*(*h*_w_ + *h*_b_) (*i—*number of reflections) should be taken into consideration to satisfy boundary conditions BC1 and BC3 and to ensure the required accuracy of calculations. Note that the values of particular currents do not change and do not depend on the number of reflections; only the values of successive reflections increase for an increasing distance of the virtual electrodes from the electric field sensor located in water.

### Four-layer model equations

All equations of the four-layer model for the considered case (Fig. 1) are derived from the relations linking the electrostatic potential V, Eq. (7), with the components of the electric field E, Eqs. (11–13), taking into account positive and negative electrodes of both types: real (Fig. 2a) and virtual (Fig. 4a–d), and the corresponding currents *I*, *I′* and *I″*, Eqs. (16) and (17).

The following equations of the electric field intensity components *E*_*x*_, *E*_*y*_, *E*_*z*_ caused by current *I* and virtual currents *I′* and *I″* for the two considered electrodes (Fig. 2a) are derived for the proposed four-layer horizontal electric current dipole model:

18 $$E_{x} = E_{xI} + E_{{xI^{\prime}}} + E_{{xI^{\prime\prime}}} ,$$

19 $$E_{y} = E_{yI} + E_{{yI^{\prime}}} + E_{{yI^{\prime\prime}}} ,$$

20 $$E_{z} = E_{zI} + E_{{zI^{\prime}}} + E_{{zI^{\prime\prime}}} ,$$

21 $${E}_{xI}=\frac{I}{4\pi {\sigma }_{w}}\left(\sum_{k=0}^{1}{\sum }_{j=0}^{1}\frac{{\left(-1\right)}^{k+1}\left(x-\left({x}_{0}+a{\left(-1\right)}^{k}\right)\right)}{{\left({\left(x-\left({x}_{0}+a{\left(-1\right)}^{k}\right)\right)}^{2}+{\left(y-{y}_{0}\right)}^{2}+{\left(z+{\left(-1\right)}^{j}h\right)}^{2}\right)}^\frac{3}{2}}\right),$$

22 $$E_{{xI^{\prime}}} = \frac{I}{{4\pi \sigma_{w} }}\frac{{\sigma_{w} - \sigma_{b} }}{{\sigma_{w} + \sigma_{b} }}\left( {\mathop \sum \limits_{i = 1}^{n} \mathop \sum \limits_{j = 0}^{1} \mathop \sum \limits_{k = 0}^{1} \mathop \sum \limits_{l = 0}^{1} \frac{{\left( { - 1} \right)^{j + 1} \left( {x - \left( {x_{0} + a\left( { - 1} \right)^{j} } \right)} \right)}}{{\left( {\left( {x - \left( {x_{0} + a\left( { - 1} \right)^{j} } \right)} \right)^{2} + \left( {y - y_{0} } \right)^{2} + \left( {z + \left( { - 1} \right)^{k} \left( {2h_{w} i + h\left( { - 1} \right)^{l} } \right)} \right)^{2} } \right)^{\frac{3}{2}} }}} \right),$$

23 $$E_{{xI^{\prime\prime}}} = \frac{I}{{4\pi \sigma_{w} }}\frac{{2\sigma_{b} }}{{\sigma_{w} + \sigma_{b} }}\left( {\mathop \sum \limits_{i = 1}^{n} \mathop \sum \limits_{j = 0}^{1} \mathop \sum \limits_{k = 0}^{1} \mathop \sum \limits_{l = 0}^{1} \frac{{\left( { - 1} \right)^{j + 1} \left( {x - \left( {x_{0} + a\left( { - 1} \right)^{j} } \right)} \right)}}{{\left( {\left( {x - \left( {x_{0} + a\left( { - 1} \right)^{j} } \right)} \right)^{2} + \left( {y - y_{0} } \right)^{2} + \left( {z + \left( { - 1} \right)^{k} \left( {2\left( {h_{w} + h_{b} } \right)i + h\left( { - 1} \right)^{l} } \right)} \right)^{2} } \right)^{\frac{3}{2}} }}} \right),$$

24 $${E}_{yI}=\frac{I}{4\pi {\sigma }_{w}}\left(\sum_{k=0}^{1}{\sum }_{j=0}^{1}\frac{y-{y}_{0}}{{\left({\left(x-\left({x}_{0}+a{\left(-1\right)}^{k}\right)\right)}^{2}+{\left(y-{y}_{0}\right)}^{2}+{\left(z+{\left(-1\right)}^{j}h\right)}^{2}\right)}^\frac{3}{2}}\right),$$

25 $$E_{{yI^{\prime}}} = \frac{I}{{4\pi \sigma_{w} }}\frac{{\sigma_{w} - \sigma_{b} }}{{\sigma_{w} + \sigma_{b} }}\left( {\mathop \sum \limits_{i = 1}^{n} \mathop \sum \limits_{j = 0}^{1} \mathop \sum \limits_{k = 0}^{1} \mathop \sum \limits_{l = 0}^{1} \frac{{y - y_{0} }}{{\left( {\left( {x - \left( {x_{0} + a\left( { - 1} \right)^{j} } \right)} \right)^{2} + \left( {y - y_{0} } \right)^{2} + \left( {z + \left( { - 1} \right)^{k} \left( {2h_{w} i + h\left( { - 1} \right)^{l} } \right)} \right)^{2} } \right)^{\frac{3}{2}} }}} \right),$$

26 $$E_{{yI^{\prime\prime}}} = \frac{I}{{4\pi \sigma_{w} }}\frac{{2\sigma_{b} }}{{\sigma_{w} + \sigma_{b} }}\left( {\mathop \sum \limits_{i = 1}^{n} \mathop \sum \limits_{j = 0}^{1} \mathop \sum \limits_{k = 0}^{1} \mathop \sum \limits_{l = 0}^{1} \frac{{y - y_{0} }}{{\left( {\left( {x - \left( {x_{0} + a\left( { - 1} \right)^{j} } \right)} \right)^{2} + \left( {y - y_{0} } \right)^{2} + \left( {z + \left( { - 1} \right)^{k} \left( {2\left( {h_{w} + h_{b} } \right)i + h\left( { - 1} \right)^{l} } \right)} \right)^{2} } \right)^{\frac{3}{2}} }}} \right),$$

27 $${E}_{zI}=\frac{I}{4\pi {\sigma }_{w}}\left(\sum_{k=0}^{1}{\sum }_{j=0}^{1}\frac{z+{\left(-1\right)}^{j}h}{{\left({\left(x-\left({x}_{0}+a{\left(-1\right)}^{k}\right)\right)}^{2}+{\left(y-{y}_{0}\right)}^{2}+{\left(z+{\left(-1\right)}^{j}h\right)}^{2}\right)}^\frac{3}{2}}\right),$$

28 $$E_{{zI^{\prime}}} = \frac{I}{{4\pi \sigma_{w} }}\frac{{\sigma_{w} - \sigma_{b} }}{{\sigma_{w} + \sigma_{b} }}\left( {\mathop \sum \limits_{i = 1}^{n} \mathop \sum \limits_{j = 0}^{1} \mathop \sum \limits_{k = 0}^{1} \mathop \sum \limits_{l = 0}^{1} \frac{{z + \left( { - 1} \right)^{k} \left( {2h_{w} i + h\left( { - 1} \right)^{l} } \right)}}{{\left( {\left( {x - \left( {x_{0} + a\left( { - 1} \right)^{j} } \right)} \right)^{2} + \left( {y - y_{0} } \right)^{2} + \left( {z + \left( { - 1} \right)^{k} \left( {2h_{w} i + h\left( { - 1} \right)^{l} } \right)} \right)^{2} } \right)^{\frac{3}{2}} }}} \right),$$

29 $$E_{{zI^{\prime\prime}}} = \frac{I}{{4\pi \sigma_{w} }}\frac{{2\sigma_{b} }}{{\sigma_{w} + \sigma_{b} }}\left( {\mathop \sum \limits_{i = 1}^{n} \mathop \sum \limits_{j = 0}^{1} \mathop \sum \limits_{k = 0}^{1} \mathop \sum \limits_{l = 0}^{1} \frac{{z + \left( { - 1} \right)^{k} \left( {2\left( {h_{w} + h_{b} } \right)i + h\left( { - 1} \right)^{l} } \right)}}{{\left( {\left( {x - \left( {x_{0} + a\left( { - 1} \right)^{j} } \right)} \right)^{2} + \left( {y - y_{0} } \right)^{2} + \left( {z + \left( { - 1} \right)^{k} \left( {2\left( {h_{w} + h_{b} } \right)i + h\left( { - 1} \right)^{l} } \right)} \right)^{2} } \right)^{\frac{3}{2}} }}} \right),$$

where: *x*_*0*_ and *y*_*0*_ are the displacements of the electrodes relative to the center of the Cartesian coordinate system in the *xy* plane, *n* denote the number of reflections, *j*, *k* and *l* denote the coefficients for subsequent reflections related to change of sign in appropriates formulas (position of virtual electrodes above/below the mirror plane, for the considered boundary between the appropriate layers—Fig. 4a–d).

A characteristic feature of the four-layer model presented above is the possibility of changing its structure (Eqs. 18–29) depending on the assumed values of parameters. Thus, e.g. if the bottom electric conductivity *σ*_*b*_ is equal to water conductivity *σ*_*w*_, the four-layer model is reduced to the three-layer model (air, water and non-conductive layers—without bottom layer), then the currents *I*′ = 0 and *I*″ = *I*, and the boundary condition BC3 is met. If, e.g. the bottom electric conductivity *σ*_*b*_ and the water conductivity *σ*_w_ are different but *h*_*b*_ tends to zero, the four-layer model is also reduced to the three-layer model (air, water and non-conductive layers). The expressions in parentheses in formulas (22, 23), (25, 26) and (28, 29) become the same and the sums in each formula give the same component in front of parentheses, formulas (21), (24) and (27), and then *I*′ + *I*″ = *I*. The boundary conditions BC1 and BC3 are also met in this case.

**Figure 5:**
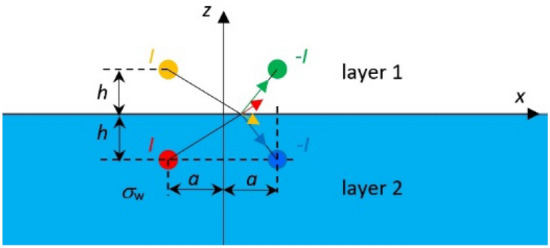
Case of two-layer model.

On the other hand, if the seabed is very deep, the four-layer model is reduced to the two-layer model (air and water layers—without bottom and non-conductive layers), then only two electrodes with current *I* (both for positive and negative electrodes) exist in the model, without any additional virtual electrodes and currents. In this case, only three Eqs. (21), (24) and (27) govern the four-layer model (the number of mirror images is equal to zero, *n* = 0) and are finally considered in the two-layer model. Hence, only four source currents generate the electric field in water (Fig. 5). Notice that the mentioned above model structure is also valid when the depth of water *h*_*w*_ is large enough, for example *h*_*w*_ = 100 m while the depth of sensor is 10 m (*z* = − 10 m), and the depth of electrodes *h* is 1 m. In this case the use of additional mirror reflections (*n* ≥ 1) is unnecessary.

### Determining the number of mirror reflections

The number of additional mirror reflections becomes crucial when electric sensors are near the bottom with lower electrical conductivity then that of seawater: *σ*_b_ < *σ*_w_. Hence, it is reasonable to ask how many reflections should be taken into account in these conditions, particularly in shallow water, to achieve satisfactory model accuracy. To answer this question, a series of simulation tests with the four-layer model and changing number of reflections *n* were carried out. Generally, the number of reflections is selected based on the iterative process. For every new analyzed scenario (new values of water and bottom depth, electric conductivity *σ*_w_ and *σ*_b_, depth of current electrodes and depth of an electric sensor), the number of reflections should be suitably increased to ensure the required accuracy of calculations.

In these tests, the reference number of *n* = 10 reflections was assumed, and then the appropriate values of dimensionless components of electric field intensity *E*_*x*_ (related to its maximal and minimal values) and *E*_*z*_ (related to its maximal value) for n = 1, …, 10 were calculated as:

30 $${\delta E}_{xmax,n}=\frac{{E}_{xmax|n}}{{E}_{{x}_{max}|n=10}},$$

31 $${\delta E}_{xmin,n}=\frac{{E}_{xmin|n}}{{E}_{{x}_{min}|n=10}},$$

32 $${\delta E}_{zmax,n}=\frac{{E}_{zmax|n}}{{E}_{{z}_{max}|n=10}}.$$

**Figure 6:**
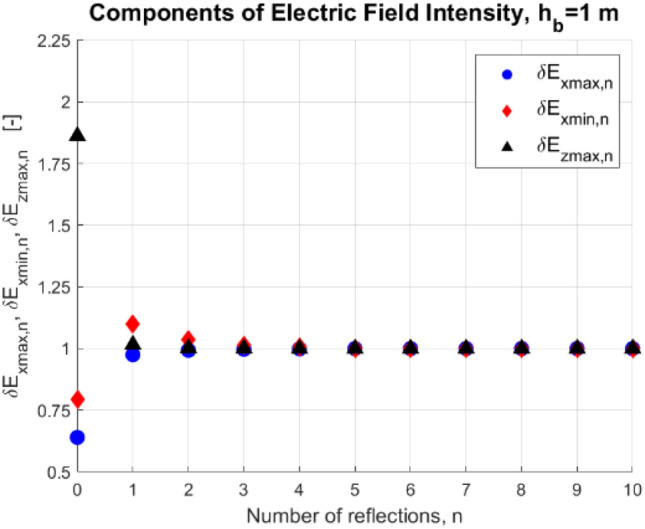
Dimensionless components of electric field intensity (*h*_w_ = 10 m, *h*_b_ = 1 m, *z* = − 9 m, *n*—number of reflections).

**Figure 7:**
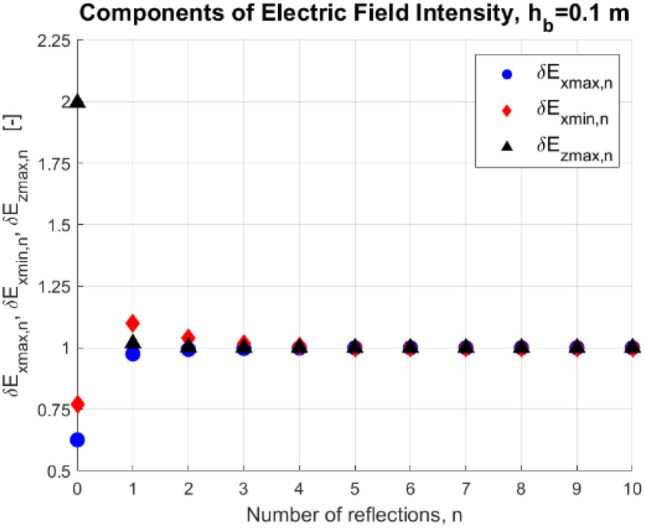
Dimensionless components of electric field intensity (*h*_w_ = 10 m, *h*_b_ = 0.1 m, *z* = − 9 m, *n*—number of reflections).

The values of dimensionless components of the electric field intensity (Eqs. 30–32) are presented in Figs. 6 and 7 as functions of *n* for water depth *h*_*w*_ = 10 m, and bottom depths *h*_*b*_ = 1 m and 0.1 m. Additionally, it was assumed that the electric conductivity of water *σ*_w_ and bottom *σ*_b_ was equal to 4 S/m and 0.4 S/m, respectively, the electrodes with source current equal to 1 A were placed at depth *h* = 1 m, and the distance between the electrodes *2a* was equal to 1 m. Notice that in this experiment, the electrodes were placed symmetrically to the center of the considered Cartesian coordinate system (*x*-axis), and the y-coordinates of both electrodes were equal to 0 (*y* = 0). That is why only the dimensionless components of electric field intensity (Eqs. 30–32) were considered. For this symmetrical electrode arrangement, the minimal and maximal values of the electric field intensity component *E*_*y*_ below the electrodes are both equal to zero (along the *z*-axis and with coordinates *x* = 0 and *y* = 0), while the minimal and maximal values of component *E*_*z*_ are equal to each other.

If we take into account only two layers (air and water, *n* = 0), then the errors in electric field intensity calculations can reach even 100% (Figs. 6 and 7), while for the four-layer model and the number of mirror reflections *n* equal to 1 the observed error in these calculations does not exceed 15%, and when *n* = *2* the maximum error drops to 5%. If the number of mirror reflections *n* is equal to 4, the relative difference of electric field intensity in relation to the case of 5 reflections is less than 0.5% (Figs. 6 and 7). Therefore for the examined case, the number of reflections *n* = 5 is considered sufficient.

Notice that for smaller water depth, the number of considered reflections is more significant.

## Verification of the analytical four-layer model—comparing to numerical FEM and BEM models

The correctness and accuracy of the analytical model presented in “Analytical model of four-layer horizontal electric current dipole” was verified and validated by comparing its results to those obtained from two numerical models of horizontal electric current dipole, which were built with the finite element method (FEM) and the boundary element method (BEM). This comparison included the analytical model structure consisting of three layers (“Case I—three-layer analytical model”, Case I) and four layers (“Case II—four-layer analytical model”, Case II).

**Figure 8:**
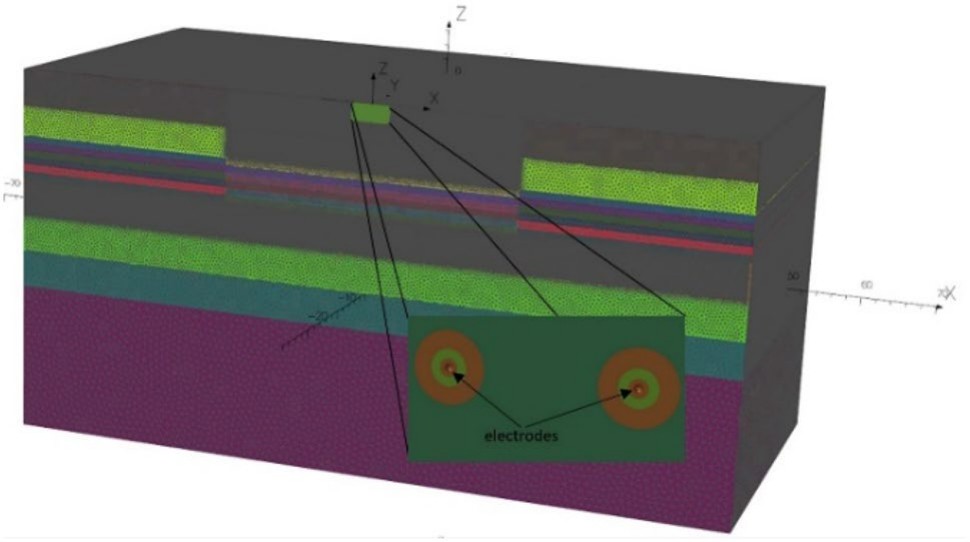
The numerical FEM model built in the Opera simulation software.

The FEM models were built in the Opera simulation software^37^ (CST Simulia Opera 2020 build 13, Professional Edition). The area of 100 m × 100 m × 50 m was taken as the test field, and the tangential electric field boundary condition was assumed at the field boundaries. The air area was not modelled in the FEM models. The area along the z-axis was divided into twelve sub-areas (sub-layers) with various thicknesses to carry out successive numerical experiments efficiently. From top to bottom, the first layer was 5 m thick, the second was 4 m thick, and the subsequent six layers were 1 m thick each. The ninth to tenth layers were 5 m thick each, and the final twelfth layer was 20 m thick. The conductivity parameters of those sub-areas (sub-layers) were changed in such a way as to obtain finally different thicknesses of the water layer, the bottom layer, and the non-conductive layer in the analyzed numerical model, without introducing additional changes in the numerical model mesh. The model built in the Opera environment is shown in Fig. 8. Different colors of the defined horizontal layers refer to different densities of the numerical grid. The total number of finite elements is enormous and amounts to 24 million, therefore only half of the area has been modelled, taking advantage of problem symmetricity about the *xz* surface.

**Figure 9:**
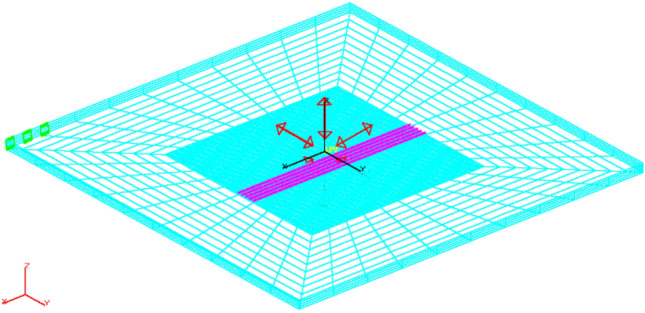
The numerical BEM model built in the SAES software package.

The BEM models were built in the simulation software developed by SAES^38,39^. In the BEM method, the air and water areas do not need to be modelled, and therefore the entire area is modelled as an empty box, thus reducing the number of elements. In BEM, the length of the limits is conditioned by the length of sources and the value of estimated electric field. For this study, the spatial limits of seawater were modelled using a box with dimensions of 100 × 100 m and the height equal to water depth (variable parameter in subsequent experiments). The air–water boundary was modelled by means of a horizontal plane of symmetry defined at depth equal to 0 m, boundary between water and air. The contour of the seawater area, close to the bottom, was meshed using 6960 elements with a different grading, increased the number of elements in the area close to the position of the source and measurement points. The horizontal plane of symmetry was not meshed. The monopoles were modelled as point sources with positive (anodes) or negative (cathodes) currents. All these elements and sources were defined inside of only one zone for the two-layer models (air and water) in the BEM model. When the bottom conductivity and its thickness were taken into account in the analysis of the three-layer model (air, water, and bottom), another zone had to be defined for the bottom in the model already including two zones. For this analysis, the bottom was modelled as a box with the same dimensions than the water model and the height equal to bottom thickness, and meshed with 200 equally spaced elements. The boundary condition defined for both models was zero current density and voltage at one element of the end of the box. Figure 9 shows the BEM model and meshing for the case under study. Red arrows in Fig. 9 indicate positions and strength directions of the monopoles, green symbols denote the boundary conditions, purple points are the measurement points, and cyan areas represent individual mesh elements.

It is essential to point out that each change in scenario characteristics (seabottom thickness or conductivity, seawater depth and/or conductivity, positions of measurement points and electric sources strength) of the FEM or BEM models leads to new numerical models which have to be built in each software environment and solved again to obtain new results for the electric field components. All these operations take incomparably much more time than the calculations making use of the four-layer analytical model (calculations have been carried out in Matlab 2015a^44^) proposed in “Analytical model of four-layer horizontal electric current dipole” (see Table 1). The calculations were carried out on the computer with two processors (12-core Intel Xeon X5660 2.8 GHz—24 logic processor) and 96 GB of RAM. The calculation time depended linearly on the number of reflections for the analytical four-layer model. For ten reflections, the calculation time was significantly less than 1 s. That means that the results were obtained much faster than in the case of calculations performed in specialized environments based on numerical FEM or BEM models.

**Table 1:** Calculation time of the analytical model and numerical FEM and BEM models.

Model	Action	Time (s)
Analytical four-layer model ("Analytical model of four-layer horizontal electric current dipole")	Calculations (for ten mirror reflections, *n* = *10*)	1 ≪
Opera (FEM)	Preparation file for calculations and execution of calculations	≈15,000
SAES (BEM)	Preparation file for calculations and execution of calculations	≈4140

### Case I—three-layer analytical model

In the first set of tests, an ‘infinite’ bottom thickness was assumed, and the analytical four-layer model was practically reduced to the three-layer model (the fourth non-conductive layer is irrelevant to the analysis carried out). The parameters of the analytical model used in this comparison analysis are shown in Table 2.

**Table 2:** Parameters of the analytical model in comparison analysis—Case I.

Symbol	Quantity	Value
*σ*_*w*_	Electric conductivity of seawater	4.0 S/m
*σ*_*b*_	Electric conductivity of bottom	0.4 S/m
*a*	Distance between electrodes	0.5 m
*h*	Electrodes depth	1.0 m
*h*_*s*_	Sensor depth	7.0 m
*h*_b_	Bottom thickness	∞ m
*h*_w_	Water depth	9.0 m

**Figure 10:**
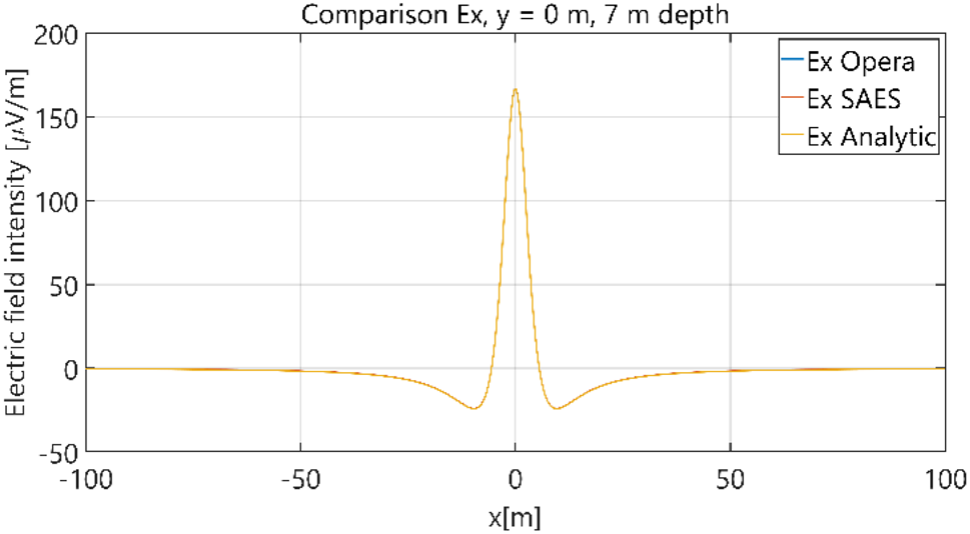
Distribution of *E*_x_ component (*y* = 0 m, *z* = − 7 m, case I).

**Figure 11:**
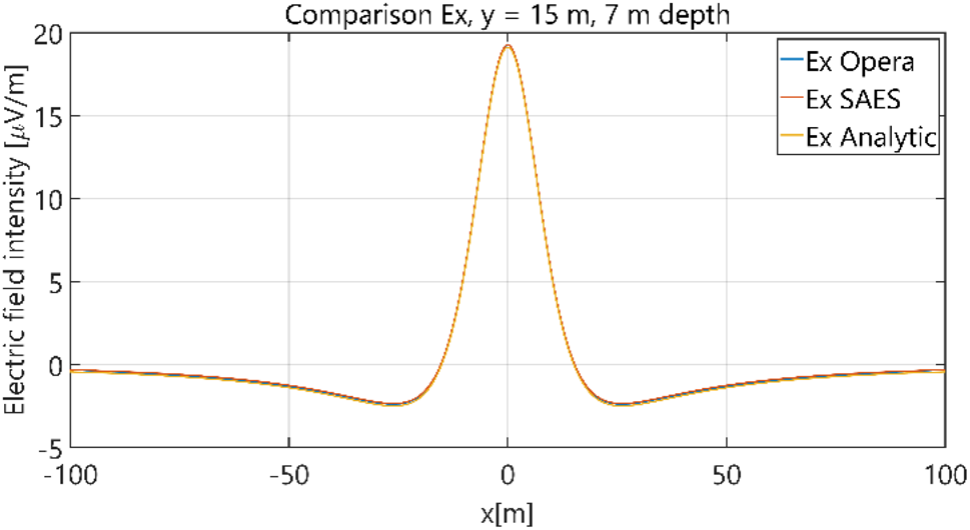
Distribution of *E*_x_ component (*y* = 15 m, *z* = − 7 m, case I).

**Figure 12:**
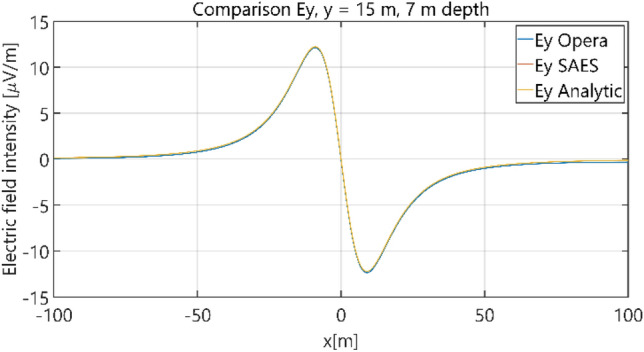
Distribution of *E*_y_ component (*y* = 15 m, *z* = − 7 m, case I).

**Figure 13:**
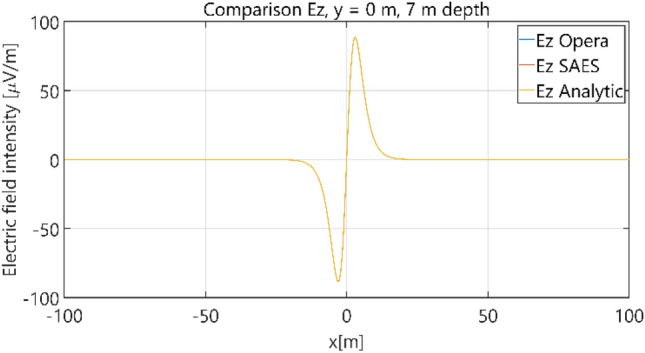
Distribution of *E*_z_ component (*y* = 0 m, *z* = − 7 m, case I).

**Figure 14:**
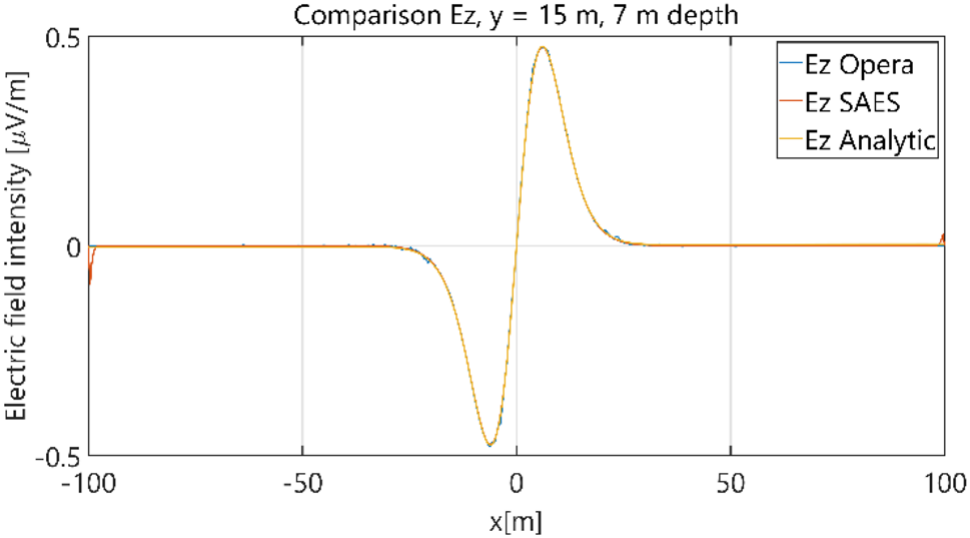
Distribution of *E*_z_ component (*y* = 15 m, *z* = − 7 m, case I).

**Figure 15:**
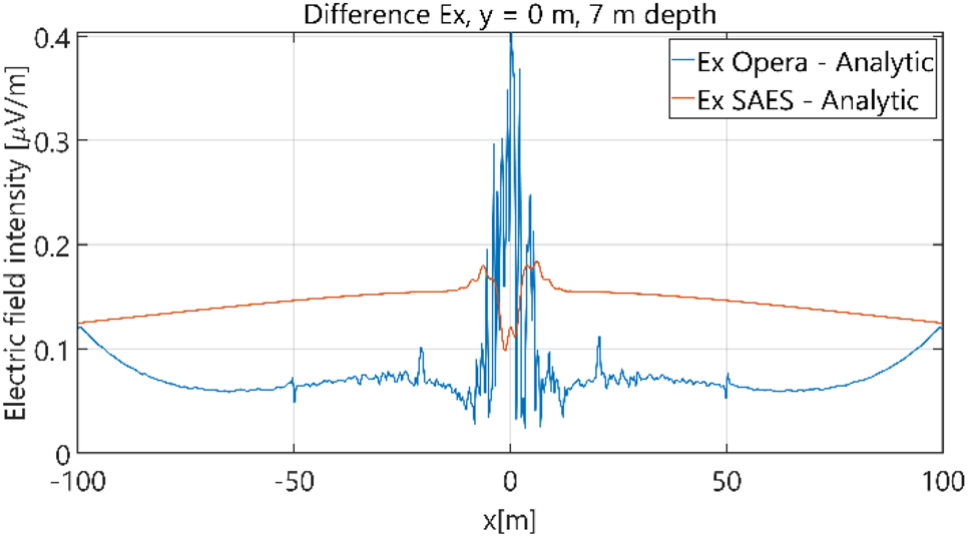
Distribution of differences between *E*_x_ values obtained from Opera (FEM) and SAES (BEM) models and the analytical model (*y* = 0 m, *z* = − 7 m).

**Figure 16:**
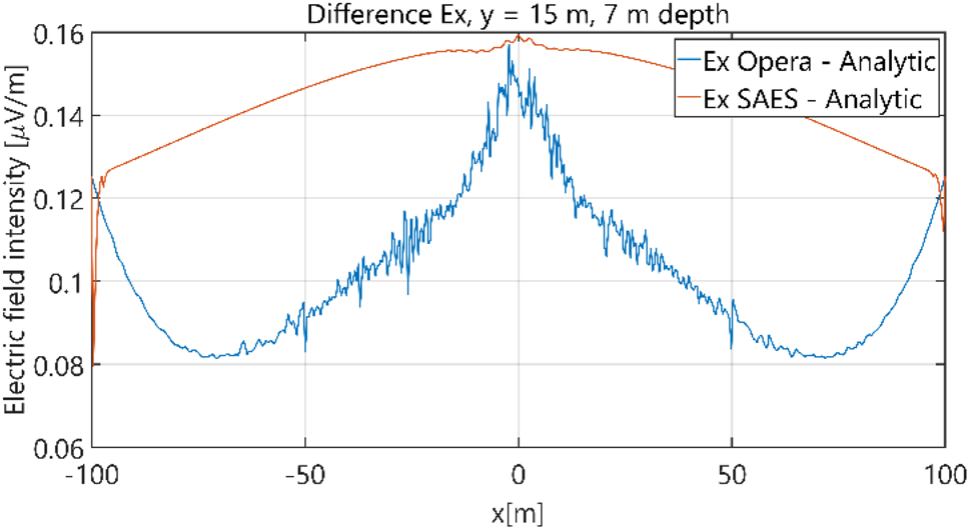
Distribution of differences between *E*_x_ values obtained from Opera (FEM) and SAES (BEM) models and the analytical model (*y* = 15 m, *z* = − 7 m, case I).

**Figure 17:**
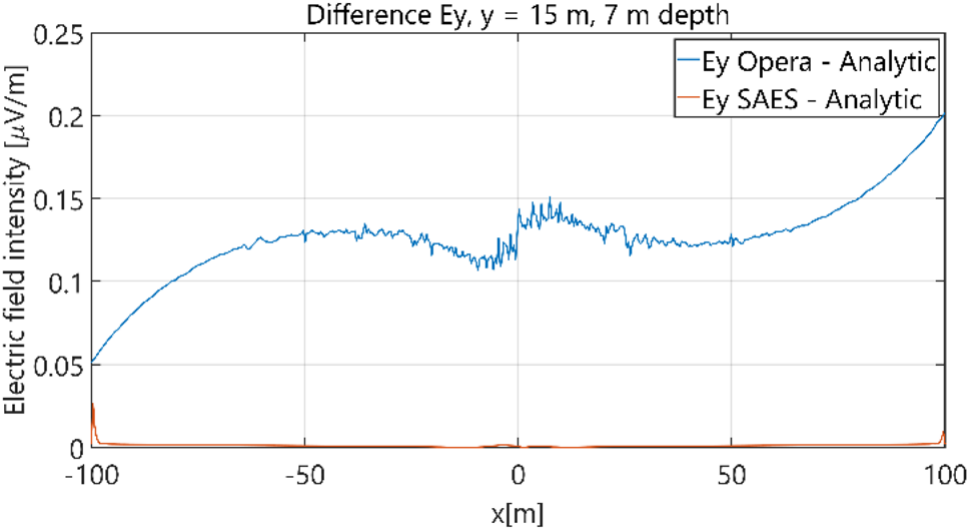
Distribution of differences between *E*_y_ values obtained from Opera (FEM) and SAES (BEM) models and the analytical model (*y* = 15 m, *z* = − 7 m, case I).

**Figure 18:**
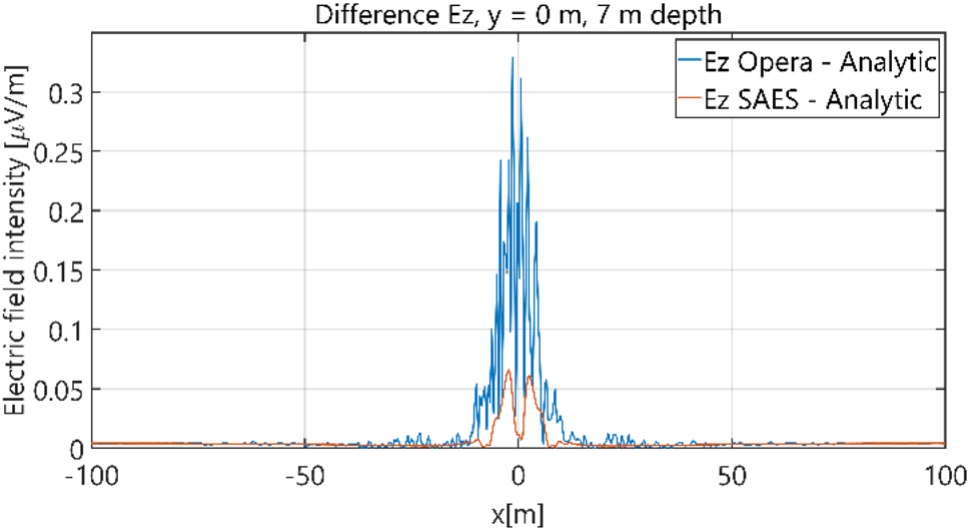
Distribution of differences between *E*_z_ values obtained from Opera (FEM) and SAES (BEM) models and the analytical model (*y* = 0 m, *z* = − 7 m, case I).

**Figure 19:**
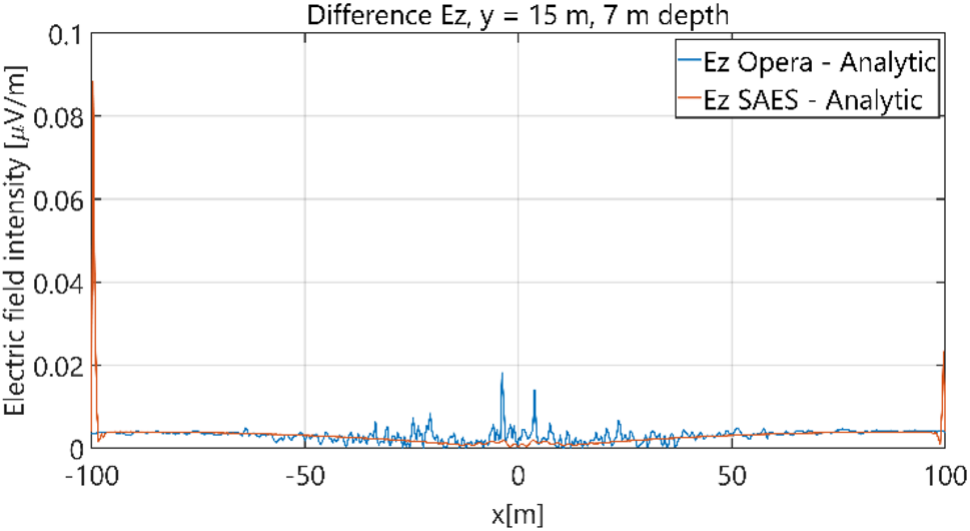
Distribution of differences between *E*_z_ values obtained from Opera (FEM) and SAES (BEM) models and the analytical model (*y* = 15 m, *z* = − 7 m, case I).

The distributions of three components of the electric field intensity (Figs. 10, 11, 12, 13 and 14) and differences between the results obtained from the analytical three-layer model and two numerical FEM and BEM models are shown in Figs. 15, 16, 17, 18 and 19 for *y* = 0 m and *y* = 15 m. For *y* = 0 m, the component *E*_y_ of the electric field intensity is equal to zero due to system symmetry and therefore is not presented in separate figures, nor is it noted in Table 3. It can be seen that the distributions of individual electric field components for all three analyzed models almost precisely coincide (Figs. 10, 11, 12, 13 and 14). Relative differences between the results of the analytical three-layer model and the numerical FEM and BEM models are shown in Table 3. They are less than 3.9% for the Opera model (FEM) and practically less than 0.85% for the SAES model (BEM) if we reject the differences on the border *x* =  ± 100 m (the results of the analytical model were taken here as the reference values). The errors on the border of the SAES numerical model (BEM) shown in Fig. 19 should be omitted because they are due to the errors of the model on the considered borders. Considering that the numerical models have limited precision, it can be concluded that the analytical model is correct and reproduces with satisfactory accuracy the electric field intensity components for the three-layer case.

**Table 3:** Relative differences between results of the analytical (three-layer) model and numerical FEM and BEM models—Case I.

Numerical model	Components of electric field	Relative difference (%)
Opera (FEM)	*E*_x_ (*y* = 0 m)	0.25
	*E*_z_ (*y* = 0 m)	0.38
	*E*_x_ (*y* = 15 m)	0.82
	*E*_y_ (*y* = 15 m)	1.71
	*E*_z_ (*y* = 15 m)	3.90
SAES (BEM)	*E*_x_ (*y* = 0 m)	0.10
	*E*_z_ (*y* = 0 m)	0.07
	*E*_x_ (*y* = 15 m)	0.85
	*E*_y_ (*y* = 15 m)	0.21
	*E*_z_ (*y* = 15 m)	18.75 0.42 (x = 0 m)

The next subsection analyses the electric field intensity results regarding the electric conductivity of the water and bottom, the depth of the water, and the thickness of the bottom. This analysis was carried out using the full analytical four-layer model.

### Case II—four-layer analytical model

The second set of tests increases the complexity of the analysis. In these tests, the water depth and the bottom depth were assumed finite (Fig. 3). The parameters of the analytical model used in this comparison analysis are shown in Table 4.

**Table 4:** Parameters of the analytical model in comparison analysis—Case II.

Symbol	Quantity	Value
*σ*_*w*_	Electric conductivity of seawater	4.0 S/m
*σ*_*b*_	Electric conductivity of bottom	0.4 S/m
*a*	Distance between electrodes	0.5 m
*h*	Electrodes depth	1.0 m
*h*_*s*_	Sensor depth	7.0 m
*h*_b_	Bottom thickness	1.0 m
*h*_w_	Water depth	9.0 m

**Figure 20:**
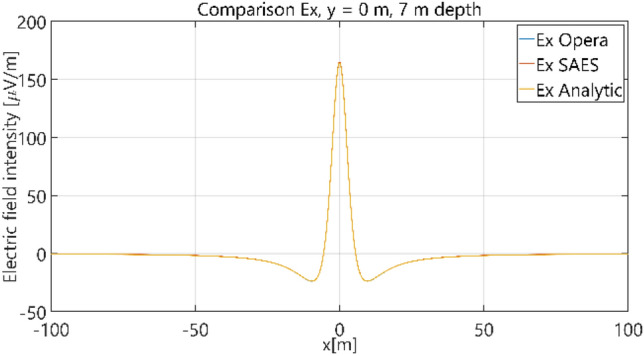
Distribution of *E*_x_ component (*y* = 0 m, *z* = − 7 m, case II).

**Figure 21:**
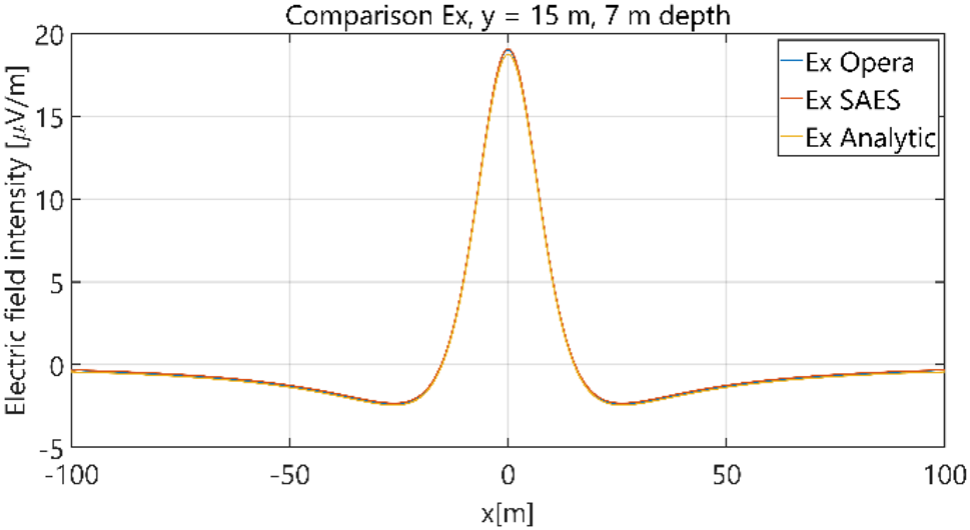
Distribution of *E*_x_ component (*y* = 15 m, *z* = − 7 m, case II).

**Figure 22:**
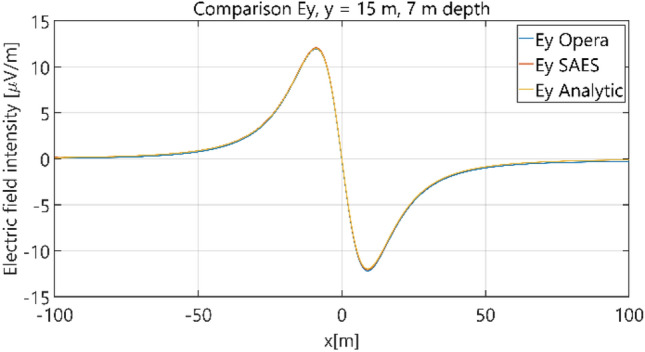
Distribution of *E*_y_ component (*y* = 15 m, *z* = − 7 m, case II).

**Figure 23:**
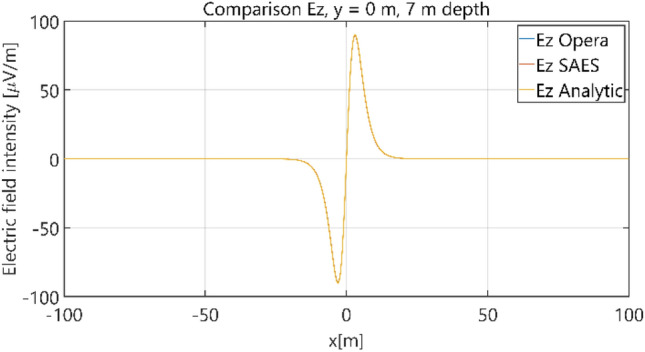
Distribution of *E*_z_ component (*y* = 0 m, *z* = − 7 m, case II).

**Figure 24:**
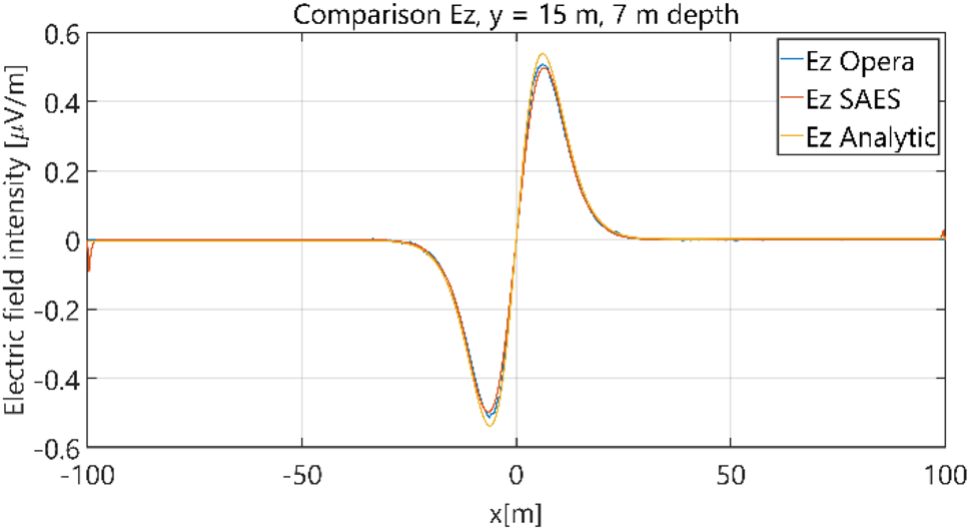
Distribution of *E*_z_ component (*y* = 15 m, *z* = − 7 m, case II).

**Figure 25:**
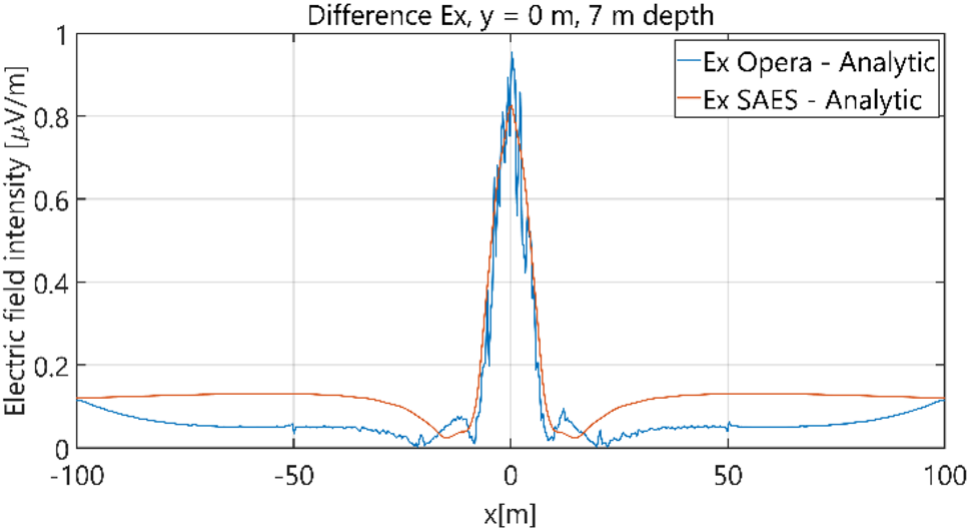
Distribution of differences between *E*_x_ values obtained from Opera (FEM) and SAES (BEM) models and the analytical model (*y* = 0 m, *z* = − 7 m, case II).

**Figure 26:**
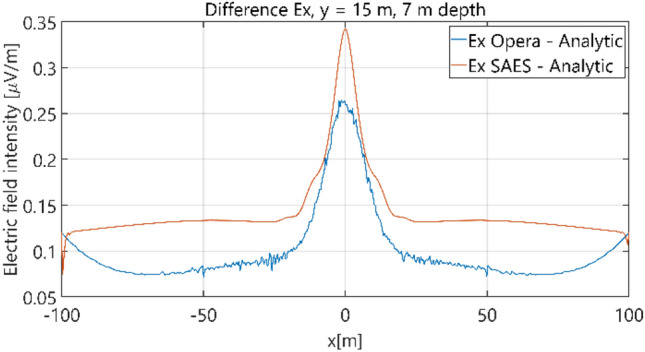
Distribution of differences between *E*_x_ values obtained from Opera (FEM) and SAES (BEM) models and the analytical model (*y* = 15 m, *z* = − 7 m, case II).

**Figure 27:**
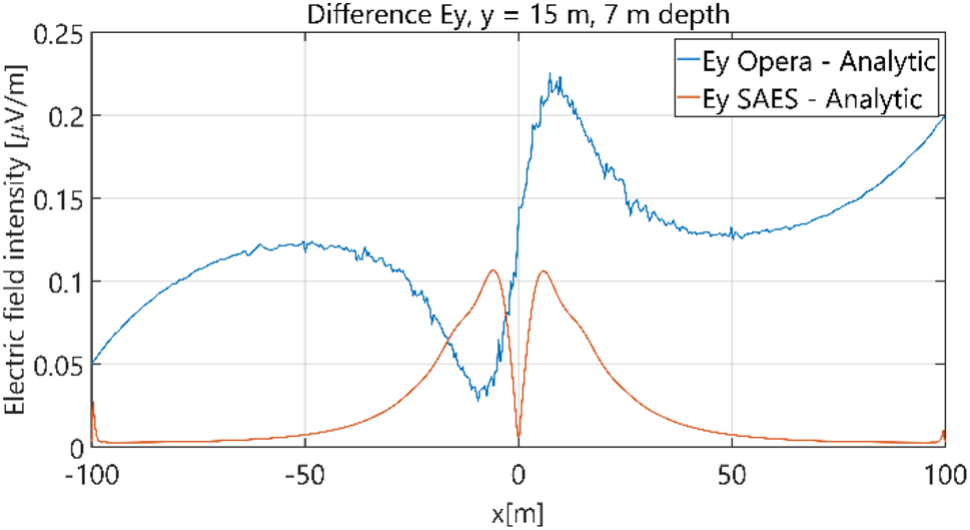
Distribution of differences between *E*_y_ values obtained from Opera (FEM) and SAES (BEM) mdoels and the analytical model (*y* = 15 m, *z* = − 7 m, case II).

**Figure 28:**
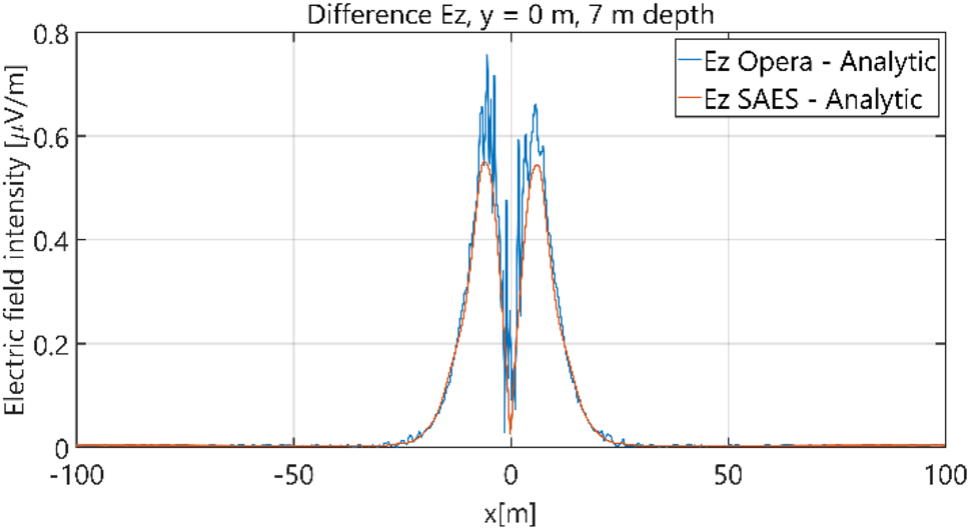
Distribution of differences between *E*_z_ values obtained from Opera (FEM) and SAES (BEM) models and the analytical model (*y* = 0 m, *z* = − 7 m, case II).

**Figure 29:**
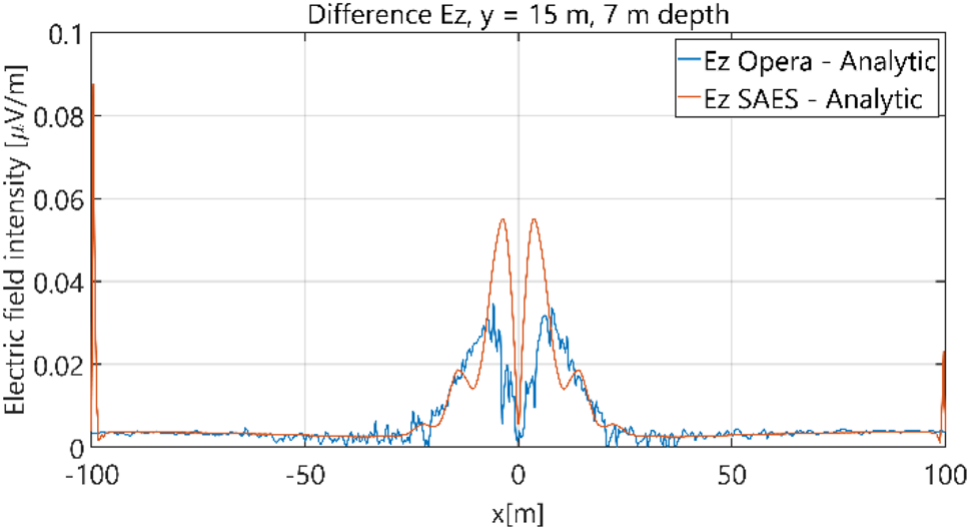
Distribution of differences between *E*_z_ valuies obtained from Opera (FEM) and SAES (BEM) models and the analytical model (*y* = 15 m, *z* = − 7 m, case II).

The distributions of three components of the electric field intensity (Figs. 20, 21, 22, 23, 24) and differences between the results obtained from the analytical four-layer model and two numerical FEM and BEM models are shown in Figs. 25, 26, 27, 28 and 29. As in the previous case, the results for y = 0 have been omitted. Also as before, it can be seen that the distributions of individual electric field components for all three analyzed models almost precisely coincide (Figs. 20, 21, 22, 23 and 24). Relative differences between the results of the analytical four-layer model and the numerical FEM and BEM models (Table 5) are less than 6% for the Opera model (FEM) and 11% (18% for *x* =  ± 100 m) for the SAES model (BEM)—except the case for *E*_z_ and *y* = 15 m presented in Fig. 29. For all other cases, relative differences are less than 2% (Table 5). Hence, it can be concluded that the analytical model is correct and reproduces with satisfactory accuracy the electric field intensity components for the four-layer case. For both the three-layer and four-layer models (“Case I—three-layer analytical model” and “Case II—four-layer analytical model”), it can be seen that the vertical component of the electric field intensity is smaller than the horizontal component, which is natural for shallow sea waters. This is related to the fact that the bottom layer's conductivity forces the current to flow ‘more’ horizontally and not penetrate ‘too much’ into seawater depth.

**Table 5:** Relative differences between results of the analytical (four-layer) model and numerical FEM and BEM models—Case II.

Numerical model	Components of electric field	Relative difference (%)
Opera (FEM)	*E*_x_ (*y* = 0 m)	0.58
	*E*_z_ (*y* = 0 m)	0.82
	*E*_x_ (*y* = 15 m)	1.40
	*E*_y_ (*y* = 15 m)	1.80
	*E*_z_ (*y* = 15 m)	6.00
SAES (BEM)	*E*_x_ (*y* = 0 m)	0.49
	*E*_z_ (*y* = 0 m)	0.60
	*E*_x_ (*y* = 15 m)	1.80
	*E*_y_ (*y* = 15 m)	0.80
	*E*_z_ (*y* = 15 m)	18.00 11.00 (x = 0 m)

**Figure 30:**
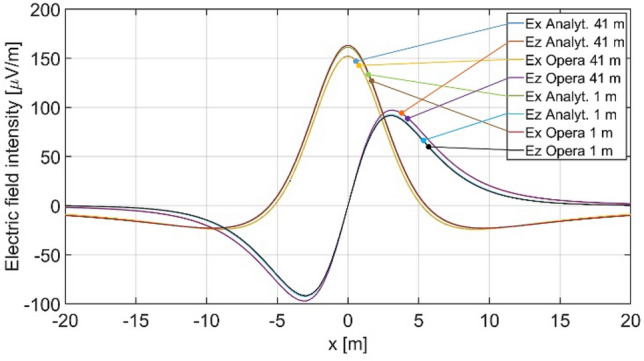
Distribution of *E*_*x*_ and *E*_*z*_ for *σ*_*w*_ = 4 S/m, *σ*_*b*_ = 1 S/m, *a* = 0.5 m, *h* = 1 m, *h*_s_ = 7 m, *h*_w_ = 9 m, *h*_b_ = 1 m (analytical and Opera—four layers) and (*h*_b_ →  ∝ analytical, h_b_ = 91 m Opera—three layers).

**Figure 31:**
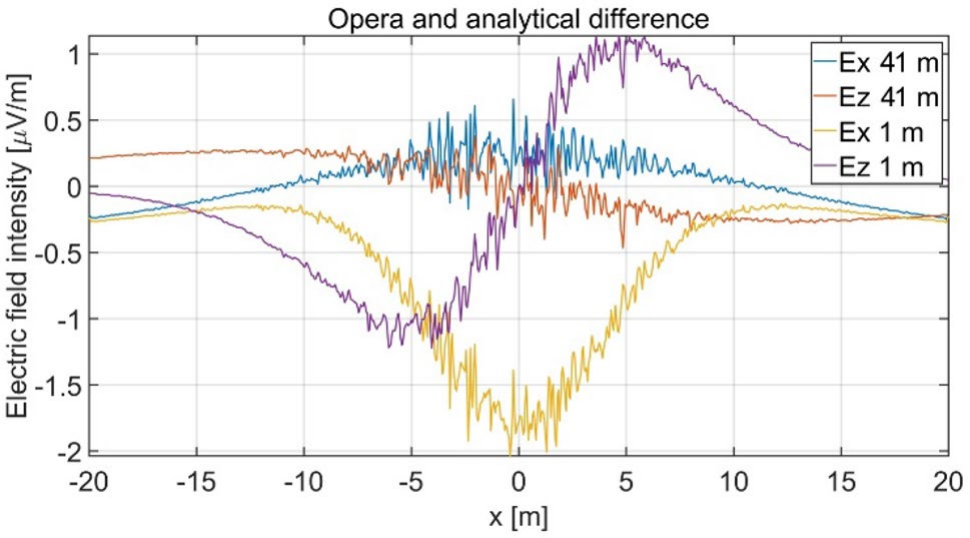
Difference of *E*_*x*_ and *E*_*z*_ between analytical and Opera solutions for three and four layers (Fig. 30).

Direct comparison the electric field intensity components generated by the analytical three-layer model and the analytical four-layer model, and the field components calculated using the Opera 3D numerical simulation environment (reference data for four layers) is presented in Figs. 30 and 31. The influence of the seabottom thickness (*h*_*b*_ = 1 m and *h*_*b*_ → ∝) on electric field (for parameters shown in Table 4) was analysed for *σ*_*b*_ = 1 S/m. The difference of *E*_*x*_ between the three-layer and four-layer model shown in Fig. 30 is over dozen percent (peak value) and *E*_*z*_ is about few percent in these cases. Figure 31 shows that differences between data of analytical and the Opera 3D numerical model are less than 2%. These results confirm the validity and significance of the analytical four-layer model proposed by the authors. A more detailed analysis of the effect of seabottom thickness on the electric field in water is presented in Paragraph 4.2—Case IV.

## Analytical four-layer model-based analysis of electric field

The previous section (“Verification of the analytical four-layer model—comparing to numerical FEM and BEM models”) has shown that the proposed analytical four-layer model is well structured and delivers results with satisfactory accuracy. This means that the proposed model can be effectively used for analyzing electric fields in seawater.

In this section, further comprehensive analyses are presented to show model’s ability to reproduce the electric field intensity in shallow and deep seawater conditions with different seabottom structure and parameters. The main objective of these analyses was to verify how the components of the electric field intensity in seawater depend on the relations between electric conductivities of water and bottom (“Case III—analysis of electric field”, Case III), and between water depth and bottom thickness (“Case IV—analysis of electric field”, Case IV).

For a clear presentation of the results obtained from a series of simulation studies, they are presented in a dimensionless form, for which it was assumed that

33 $${E}_{u{x}_{max}}=\frac{{E}_{{x}_{max}}}{{E}_{{x}_{max}|{h}_{w}\to \infty }},$$

34 $${E}_{u{x}_{min}}=\frac{{E}_{{x}_{min}}}{{E}_{{x}_{min}|{h}_{w}\to \infty }},$$

35 $${E}_{u{y}_{max}}=\frac{{E}_{{y}_{max}}}{{E}_{{x}_{max}|{h}_{w}\to \infty }},$$

36 $${E}_{u{z}_{max}}=\frac{{E}_{{z}_{max}}}{{E}_{{z}_{max}|{h}_{w}\to \infty }},$$

37 $${h}_{uw}=\frac{{h}_{w}}{10 },$$

38 $${h}_{ub}=\frac{{h}_{b}}{10 },$$

39 $${\sigma }_{u}=\frac{{\sigma }_{w}}{{\sigma }_{b}},$$

40 $${y}_{u}=\frac{y}{10},$$

where subscript *u* denotes the dimensionless value of the considered parameter, and *σ*_*u*_ is the relation between electrical conductivities of water and bottom.

When analyzing formulas (33)–(40), the electric field intensity components were related to the case of 'infinite' depth of water. The depth and distance between the electrodes were assumed equal to 1 m, and the dimensionless distance value was equal to 0.1. The dimensionless depth of the electric sensor was assumed equal to 0.9 in the performed analysis. The water depth *h*_*w*_, the bottom thickness *h*_*b*_, and the distance between the electrodes in the *y*-axis direction from the origin of the Cartesian reference system were normalized taking into account the maximal distance of 10 m.

### Case III—analysis of electric field

**Figure 32:**
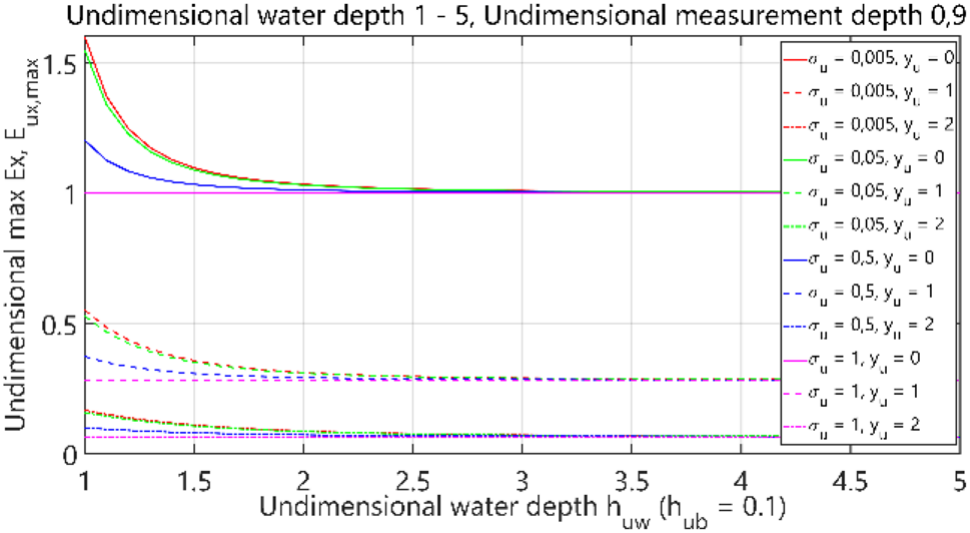
Dependence of *E*_ux,max_ on *h*_uw_ (*h*_ub_ = 0.1).

**Figure 33:**
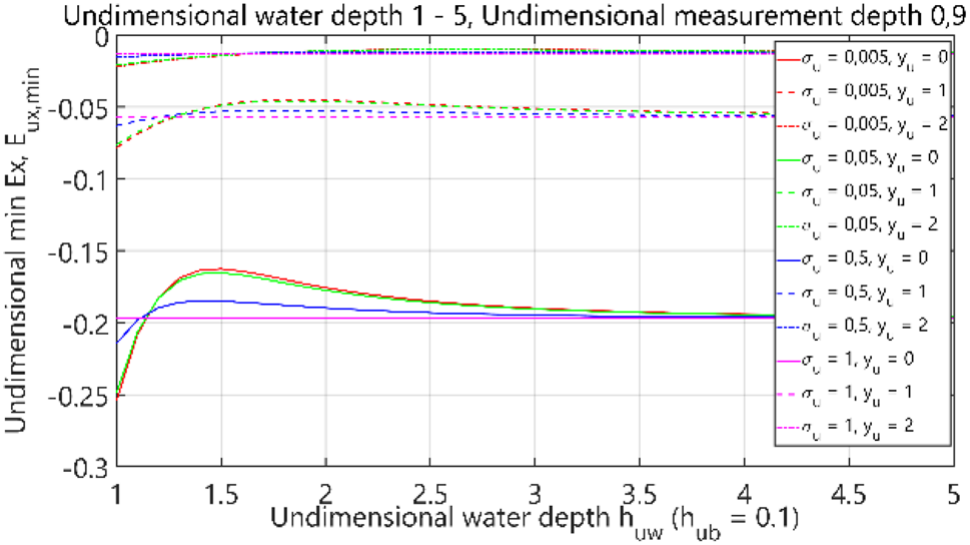
Dependence of *E*_ux,min_ on *h*_uw_ (*h*_ub_ = 0.1).

**Figure 34:**
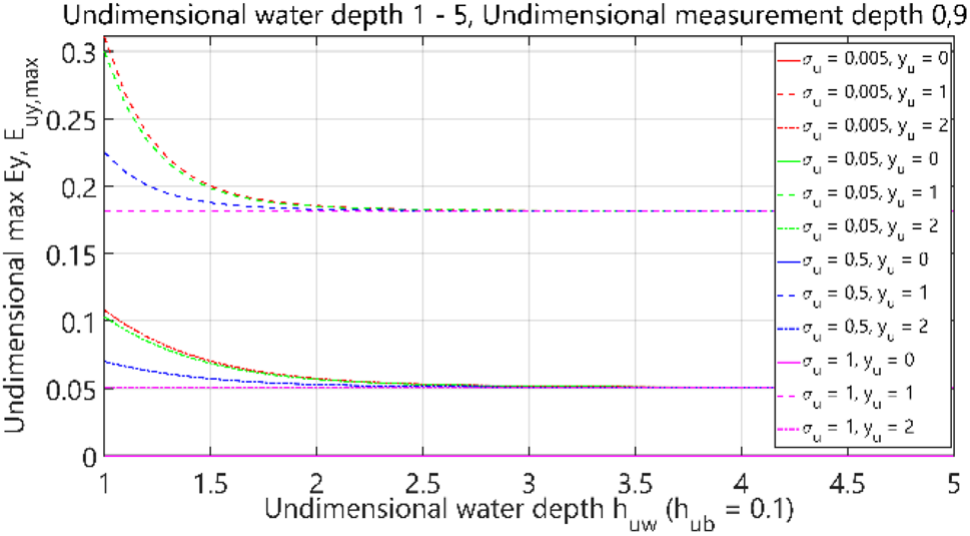
Dependence of *E*_uy,max_ on *h*_uw_ (*h*_ub_ = 0.1).

**Figure 35:**
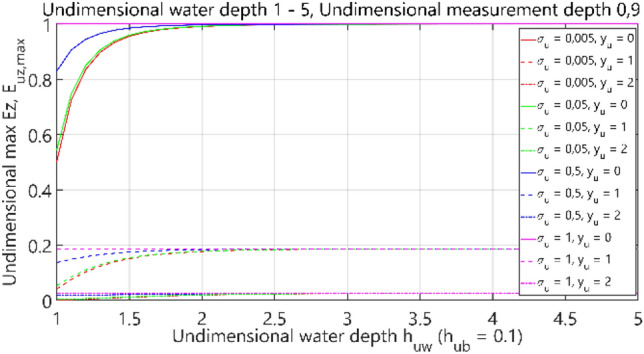
Dependence of *E*_uz,max_ on *h*_uw_ (*h*_ub_ = 0.1).

The third analysis concerned the case of 4 layers: air–water–bottom–non-conductive layer, with constant thickness of the bottom layer. The obtained results are summarised in Figs. 32, 33, 34 and 35, where each figure presents appropriately max and min dimensionless values of electric field components *E*_*x*_, *E*_*y*_ and *E*_*z*_ as waveforms covering twelve cases (four different values of dimensionless *σ*_*u*_ ∈ (0.005, 0.05, 0.5, 1) and three different values of dimensionless *y*_*u*_ ∈ (0, 1, 2)) according to the changes of dimensionless bottom thickness *h*_*uw*_. In contrast to the test results presented in “Determining the number of mirror reflections”, the maximum value of the electric field intensity component *E*_*y*_ was also included in the analysis, since in this section, the electric field intensity components were also analysed for different values of *y*_*u*_ (distance from the centre of the adopted coordinate system along the *y*-axis).

It can be seen that for the dimensionless water depth *h*_uw_ greater than 2.5 (Figs. 32, 33, 34 and 35), the dimensionless electric conductivity *σ*_u_ is negligible. It means that the electric conductivity of the bottom can be omitted when the water is much deeper than the sensor position depth. But if the water is shallow in relation to the sensor position depth, a small value of electric conductivity of the bottom increases the *E*_x_ component of electric field intensity up to 60% and decreases the *E*_z_ component down to 50%, as compared to the values for ‘infinite’ water depth (Figs. 32 and 35 for *h*_uw_ = 1). Therefore, it can be concluded that the electric conductivity of the bottom is essential, especially in shallow sea waters (*h*_*uw*_ ∈  < 1, 1.5) >).

### Case IV—analysis of electric field

**Figure 36:**
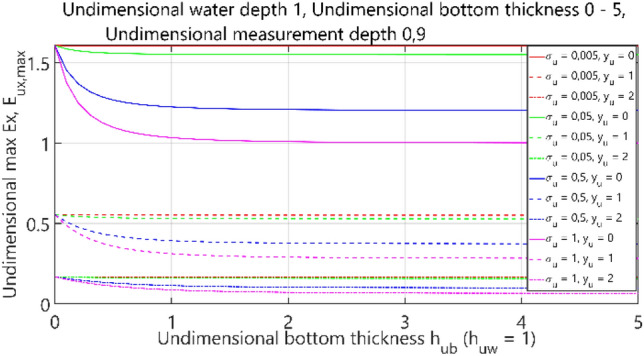
Dependence of *E*_ux,max_ on *h*_ub_.

**Figure 37:**
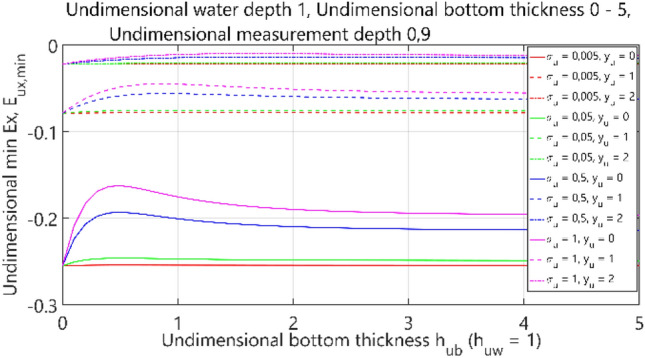
Dependence of *E*_ux,min_ on *h*_ub_.

**Figure 38:**
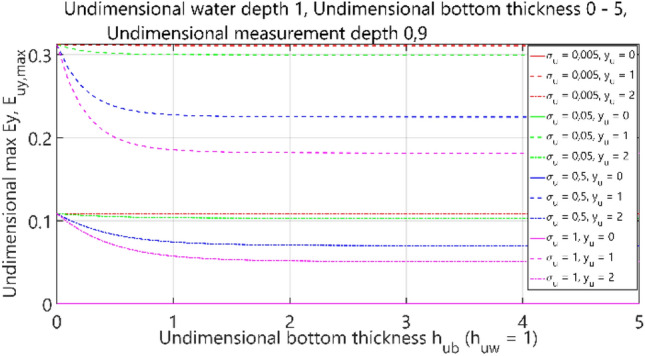
Dependence of *E*_uy,max_ on *h*_ub_.

**Figure 39:**
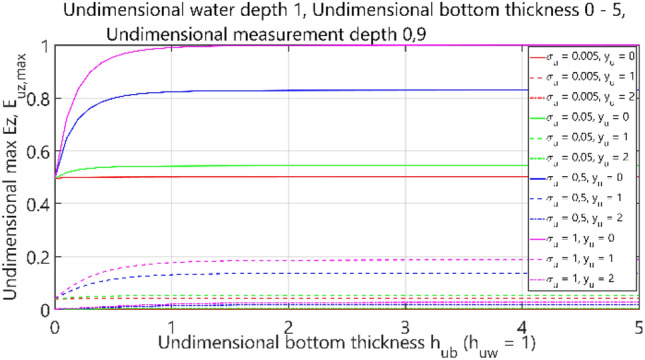
The Dependence of *E*_uz,max_ on *h*_ub_.

The fourth case of the performed analysis concerned the 4-layer model (air–water–bottom–non-conductive layer) with constant thickness of the water layer. In this set of tests, the dimensionless water depth was assumed equal to 1. The obtained results are summarised in Figs. 36, 37, 38 and 39, where each figure presents appropriately max and min values of dimensionless electric field components *E*_*x*_, *E*_*y*_ and *E*_*z*_ as waveforms covering twelve cases (four different values of dimensionless *σ*_*w*_ ∈ (0.005, 0.05, 0.5, 1) and three different values of dimensionless *y*_*u*_ ∈ (0, 1, 2)) according to the changes of dimensionless bottom thickness *h*_*ub*_.

It can be seen that for the dimensionless botom depth equal to 0, the electric field intensity component *E*_x_ is larger by about 60%, and the component *E*_z_ is smaler by about 50% than the corresponding components in the ‘infinite’ water case (Figs. 36 and 39). It can also be observed that the electric field intensity component *E*_x_ decreases and the component *E*_z_ increases when the bottom thickness increases and the electrical conductivity of the bottom takes larger values. Generally, the increase of *E*_z_ (Fig. 39) and decrease of *E*_x_ (Figs. 36, 37) and *E*_y_ (Fig. 38) as a result of the increase of bottom depth *h*_*b*_ is more significant for higher values of bottom electric conductivity *σ*_*w*_. The differences between the values of electric field intensity components reach even as much as 60% (Fig. 36). It can be concluded that in this case the bottom thickness *h*_*b*_ is essential and has to be taken into account, especially when this thickness is relatively small and the value of bottom conductivity of *σ*_*b*_ is close to that of water conductivity *σ*_*w*_.

## Conclusions

In the technical literature, the electric field intensity is usually described using a three-layer model of horizontal electric current dipole. In the model proposed in this paper, a fourth layer is introduced to allow a more detailed and reliable analysis of the electric field, especially in shallow coastal waters. The fourth layer has special interest when the sea bottom has an estimated thickness, and it is needed to be introduce this environmental characteristic in the model. Due to the attenuation law of electromagnetic waves in seawater, the electric field decreases dramatically with the distance, therefore, the electric signatures are considered as near field signatures. Hence, the electric field propagation has especial interest in shallow coastal waters areas where the distance between the source and measurement system are limited by the water depth.

The performed analysis has shown that the proposed four-layer analytical model of horizontal electric dipole can be used for fast and reliable calculations of the electric field in seawater in the presence of the fourth layer. Furthermore, the performed simulations tests and their results have shown that the bottom thickness and its electric conductivity significantly affect to the electric field's intensity distribution in shallow seawater. The comparative analysis of the results obtained from the proposed analytical model and from FEM and BEM numerical models confirmed the correctness and accuracy of the four-layer model. The differences of results between the numerical models and the analytical model were only at the level of a few percent. The FEM and BEM numerical methods have been designed to be used from the definition phase up to the sea trials. These models can use polarization curves or point sources and due to the amount of mesh points, require large computational time to solve electric field models. For this reason, these methods cannot be used for real-time operations, being the analytical models widely used. The analytical four-layer model performs the estimations of UEP faster than the numerical methods, almost a thousand times and therefore can be used for real time operations solving models based on dipoles.

Future works will focus on the use of the presented analytical model of horizontal electrical current dipole to develop a multi-dipole model of ship's electric field which will allow reconstructing its electric signature in any maritime area. In that case, the ship's electric signature should be measured on a measuring range with known values of seawater depth, bottom thickness, and electric conductivity of seawater and bottom. Once its electric signature is known from those experiments and its multi-dipole model parameters are identified, the electric signature of the ship may be reconstructed for any maritime conditions. This kind of procedure may be used to protect ships against sea mines, or other underwater detection systems triggered by making use of electric field intensity measurements. This problem is relevant not only for naval ships but also for civilian transport vessels operating in shallow coastal waters.
