# Author Correction: An analytical four-layer horizontal electric current dipole model for analysing underwater electric potential in shallow seawater

**DOI:** 10.1038/s41598-023-46597-9

**Published:** 2023-11-07

**Authors:** Miroslaw Woloszyn, Krystian Buszman, Tomasz Rutkowski, Jaroslaw Tarnawski, Francisco Javier Rodrigo Saura

**Affiliations:** 1 Faculty of Electrical and Control Engineering, Gdansk University of Technology, Gdansk, Poland; 2 Faculty of Navigation and Naval Weapons, Polish Naval Academy, Gdynia, Poland; 3 SAES-Sociedad Anónima de Electrónica Submarina, Cartagena, Spain

Correction to: *Scientific Reports* 10.1038/s41598-022-12645-z, published online 24 May 2022

The original version of this Article contained an error in the spelling of the author Miroslaw Woloszyn which was incorrectly given as Miroslaw Wołoszyn.

The original Article has been corrected.

